# RPW8.1 enhances the ethylene‐signaling pathway to feedback‐attenuate its mediated cell death and disease resistance in *Arabidopsis*

**DOI:** 10.1111/nph.16857

**Published:** 2020-09-05

**Authors:** Zhi‐Xue Zhao, Qin Feng, Peng‐Qiang Liu, Xiao‐Rong He, Jing‐Hao Zhao, Yong‐Ju Xu, Ling‐Li Zhang, Yan‐Yan Huang, Ji‐Qun Zhao, Jing Fan, Yan Li, Shunyuan Xiao, Wen‐Ming Wang

**Affiliations:** ^1^ Rice Research Institute and Key Lab for Major Crop Diseases Sichuan Agricultural University Chengdu 611130 China; ^2^ Institute of Biosciences and Biotechnology Research & Department of Plant Science and Landscape Architecture University of Maryland College Park MD 20850 USA

**Keywords:** *ACO4*, *EIN3/EIL1*, *ERF016*, *ERF6*, ethylene signaling, *ORA59*, powdery mildew, *RPW8.1*

## Abstract

- The *Arabidopsis RESISTANCE TO POWDERY MILDEW 8.1* (*RPW8.1*) activates confined cell death and defense against different pathogens. However, the underlying regulatory mechanisms still remain elusive.
- Here, we show that RPW8.1 activates ethylene signaling that, in turn, negatively regulates *RPW8.1* expression. RPW8.1 binds and stabilizes 1‐aminocyclopropane‐1‐carboxylate oxidase 4 (ACO4), which may in part explain increased ethylene production and signaling in *RPW8.1*‐expressing plants. In return, ACO4 and other key components of ethylene signaling negatively regulate RPW8.1‐mediated cell death and disease resistance via suppressing *RPW8.1* expression.
- Loss of function in *ACO4*, *EIN2*, *EIN3 EIL1*, *ERF6*, *ERF016* or *ORA59* increases RPW8.1‐mediated cell death and defense response. By contrast, overexpression of *EIN3* abolishes or significantly compromises RPW8.1‐mediated cell death and disease resistance. Furthermore, *ERF6*, *ERF016* and *ORA59* appear to act as trans‐repressors of *RPW8.1*, with *OAR59* being able to directly bind to the *RPW8.1* promoter.
- Taken together, our results have revealed a feedback regulatory circuit connecting *RPW8.1* and the ethylene‐signaling pathway, in which *RPW8.1* enhances ethylene signaling, and the latter, in return, negatively regulates RPW8.1*‐*mediated cell death and defense response via suppressing *RPW8.1* expression to attenuate its defense activity.

## Introduction

Plants employ pathogen‐associated molecular patterns (PAMPs)‐triggered immunity (PTI) and effector‐triggered immunity (ETI) to protect themselves from the invasion of pathogens (Jones & Dangl, 2006). Upon perception of the pathogen‐derived signals, plants also activate a network of phytohormone‐mediated and defense‐related signaling pathways to limit pathogen invasion. Such a network includes activation of, and crosstalk between, salicylic acid (SA) signaling, ethylene signaling and jasmonic acid (JA) signaling. The SA‐signaling pathway is mainly involved in defense against biotrophic pathogens, while the ethylene‐ and JA‐signaling pathways are mainly involved in defense against necrotrophic pathogens (McDowell & Dangl, 2000).

Ethylene is a small gaseous hydrocarbon that regulates diverse morphological, physiological and immune responses in plants (Khan, 2005; Khan *et al*., 2008). Ethylene synthesis starts with the conversion of S‐adenosyl‐l‐methionine (SAM) into 1‐aminocyclopropane‐1‐carboxylic acid (ACC) by ACC synthases (ACS), which is the rate‐limiting step in ethylene biosynthesis (Broekaert *et al*., 2006). Subsequently, ACC oxidase (ACO) converts ACC into ethylene (Dorling & McManus, 2018). The ethylene‐signaling pathway has been well characterized through extensive molecular and genetic studies. In the absence of ethylene, a group of ethylene receptors promote the activity of the Raf‐like protein kinase CONSTITUTIVE TRIPLE RESPONSE 1 (CTR1) (Gao *et al*., 2003). Activated receptor/CTR1 complexes inhibit the membrane protein ETHYLENE INSENSITIVE2 (EIN2) through protein phosphorylation at the C‐terminal domain of EIN2 (Alonso *et al*., 1999), and subsequent degradation by the 26S proteasome proteolytic pathway (Qiao *et al*., 2009). EIN2 is a key component of the ethylene‐signaling pathway (Qiao *et al*., 2009). Functioning downstream of EIN2, two transcription factors, ETHYLENE INSENSITIVE 3 (EIN3) and EIN3‐LIKE 1 (EIL1), regulate the expression of ethylene‐responsive factors (ERFs) (Solano *et al*., 1998; Chang *et al*., 2013). In the presence of etylene, ethylene is perceived through its binding to the receptors and inhibits the activity of the receptor/CTR1 complexes. Consequently, the inhibition of EIN2 by CTR1 is relieved, and the C‐terminus of EIN2 is cleaved and translocated to the nucleus to directly or indirectly activate EIN3 and EIL1 (Ju *et al*., 2012). ERFs are transcription factors (TFs) with two main features: binding to DNA and regulating the transcription of downstream genes (Fujimoto *et al*., 2000). ERFs bind to the DNA sequences containing GCC (AGCCGCC) and/or DRE/CRT (A/GCCGAC) boxes via their APETALA2/ETHYLENE RESPONSE FACTOR (AP2/ERF) domain (Muller & Munne‐Bosch, 2015).

It has been well established that ethylene signaling and JA signaling orchestrate plant immune responses. For example, JA signaling enhances the function of EIN3 and EIL1, leading to the upregulation of *ERF1* and *ORA59* (Zhu *et al*., 2011). The ethylene‐signaling pathway has also been shown to have crosstalk with the SA signaling pathway. For example, EIN3 negatively regulates SA signaling via directly binding to the promoter of the key SA biosynthesis gene *SALICYLIC ACID INDUCTION‐DEFICIENT2* (*SID2*) and repressing its expression (Chen *et al*., 2009). On the other hand, SA strongly reduces the accumulation of OCTADECANOID‐RESPONSIVE ARABIDOPSIS AP2/ERF 59 (ORA59), an ERF TF that integrates ethylene‐ and JA‐signaling, and regulates resistance to necrotrophic pathogens (Van der Does *et al*., 2013). Consistent with an antagonistic relationship between the ethylene pathway and the SA pathway, the *ein3 eil1* double or the *ein2* single mutant constitutively accumulates SA and exhibits enhanced disease resistance to *Pseudomonas syringae* (Chen *et al*., 2009).

Interestingly, recent studies suggested that ethylene signaling is involved in positive regulation of PTI. For example, ethylene treatment induces BIK1 phosphorylation in a PEPR‐dependent manner in *Arabidopsis*, and *bik1* mutations compromise ethylene‐induced expression of defense‐related genes and resistance against *Botrytis cinerea* (Liu *et al*., 2013). EIN3 and potentially EIL1 promote the expression of *FLS2* via direct binding to the promoter of *FLS2* (Boutrot *et al*., 2010). Loss‐of‐function mutations in EIN2 result in lower sensitivity of *Arabidopsis* mutant plants to both elf18 and flg22 in different defense‐related outputs (Tintor *et al*., 2013). More recently, it was reported that ethylene waves constitute essential root immune response upon nematode infection (Marhavý *et al*., 2019).

The role of ethylene signaling in plant resistance against powdery mildew is less clear. The *Arabidopsis ein2* mutant has been shown to be slightly more susceptible to a powdery mildew pathogen, *Golovinomyces cichoracearum* UCSC1 (Xiao *et al*., 2005), implying a positive role of ethylene in basal resistance. However, powdery mildew infection causes a gradual and exponential growth in ethylene emission in the susceptible plants, but does it to a much lesser extent in the resistant plants (Harrach *et al*., 2008), implying that ethylene production is correlated with disease susceptibility. Regardless of the negative or positive role of ethylene signaling in plant immunity, *ERF*s are transcription factors that integrate ethylene signaling with the major defense pathways (Muller & Munne‐Bosch, 2015). For example, *ERF1* promotes ethylene‐inducible defense gene expression, and overexpression of *ERF1* enhances resistance to necrotrophic pathogens in *Arabidopsis* (Berrocal‐Lobo & Molina, 2004). *ERF6* is phosphorylated by MPK3/MPK6 and subsequently activates the expression of *PR* genes such as *PDF1.2*, thereby contributing to resistance against a necrotrophic fungal pathogen *B. cinerea* (Meng *et al*., 2013). Overexpression of another key *ERF*, *ORA59*, enhances resistance against *B. cinerea*, whereas knockdown of *ORA59* results in enhanced susceptibility to the same pathogen (Pré *et al*., 2008). Interestingly, *ERF016* (*AT5G21960*) was found to bind directly the GCC‐box of the *PDF1.2* promoter in a yeast one‐hybrid (Y1H) assay; however, the biological function of *ERF016* is currently unclear (Ou *et al*., 2011).

*Arabidopsis RESISTANCE TO POWDERY MILDEW 8.1* (*RPW8.1*) and *RPW8.2*, identified from accession Ms‐0, encode atypical resistance (R) proteins containing a coiled‐coil (CC) domain that shows sequence homology to the CC domain of some CC–nucleotide‐binding site–leucine‐rich repeat type R proteins (Xiao *et al*., 2001). RPW8.1 shares 45% identity and 65% similarity in amino acid sequence with RPW8.2, and both of them confer broad‐spectrum resistance against powdery mildew pathogens (Xiao *et al*., 2001). While SA signaling is required for the activation of the *RPW8.2* promoter, the *RPW8.1* promoter is constitutively expressed (Ma *et al*., 2014). RPW8.2 is partitioned to the nucleus, the cytoplasm and the extrahaustorial membrane (EHM) via multiple intramolecular trafficking signals to mediate EHM‐focused resistance to powdery mildew diseases (Wang *et al*., 2009; Kim *et al*., 2014; Huang *et al*., 2019). When expressed from its native promoter, RPW8.1 is localized to a membrane‐like structure surrounding chloroplasts in the mesophyll cells to promote basal defense against different pathogens and hypersensitive response‐like cell death (Ma *et al*., 2014; Li *et al*., 2018). Interestingly, the cell death lesions activated by expression of *RPW8.1* as a transgene in Col‐*gl* (a glabrous mutant of Col‐0) are often discrete and confined (Ma *et al*., 2014), implying that RPW8.1’s function is subjected to tight negative regulation. However, how RPW8.1‐activated cell death and defense are controlled remains unclear.

In order to understand the underlying regulatory mechanism of RPW8.1‐mediated immunity, we performed yeast two‐hybrid (Y2H) assay to screen for potential interacting proteins of RPW8.1 and found that one of the putative interactors is 1‐aminocyclopropane‐1‐carboxylate oxidase 4 (ACO4), which is one of the ACC oxidases converting ACC into ethylene. We provided further evidence to demonstrate that RPW8.1 interacts with and stabilizes ACO4. Results from our subsequent genetic and molecular analysis indicate that RPW8.1 promotes the ethylene‐signaling pathway in part via binding to and stabilizing ACO4 to promote ethylene biosynthesis and signaling. Elevated ethylene signaling, in return, attenuates RPW8.1‐mediated cell death and defense through mechanisms including transcriptional repression of *RPW8.1* via ORA59 binding to the *RPW8.1* promoter. Thus, this study reveals an autoregulatory circuit linking the ethylene‐signaling pathway with RPW8.1‐mediated immunity.

## Materials and Methods

### Plant materials and growth conditions

R1Y4 is a transgenic Col‐*gl* line (containing the *glabrous* mutation in the Col‐0 background) expressing *RPW8.1‐YFP* from the native promoter of *RPW8.1* originally identified in the *Arabidopsis* Ms‐0 accession (Xiao *et al*., 2001; Ma *et al*., 2014). The length of the *RPW8.1* promoter is 1137 bp upstream of the ATG start codon of *RPW8.1* (Ma *et al*., 2014). A transgenic Col‐*gl* line expressing RPW8.2–YFP (R2Y4) from its native promoter was from a previous study (Wang *et al*., 2009). Other *Arabidopsis* lines, including *ein2‐1* (Roman *et al*., 1995), *ein3‐1 eil1‐1* (Alonso *et al*., 2003) and the *EIN3ox* (35S:*EIN3*) transgenic line (Chen *et al*., 2009), were kindly provided by other laboratories. The *aco4* mutant is a homozygous T‐DNA insertion line (SALK_064286C) from the Arabidopsis Biological Resource Center. The various mutant alleles of the ethylene pathway genes were introduced into the R1Y4 background by genetic crossing, and confirmed by PCR‐based genotyping as previously described, resulting in the lines *aco4*/R1Y4, *ein2‐1*/R1Y4/Col‐*gl*, *ein2‐1*/Col‐*gl*, *ein3‐1 eil1‐1*/R1Y4/Col‐0, *ein3‐1 eil1‐1*/R1Y4/Col‐*gl* and *EIN3ox*/R1Y4 for genetic analysis in this study.

*Arabidopsis* seeds were sown in the peat soil (PINDSTRUP) and were cold‐treated at 4°C for 48 h to improve germination. *Arabidopsis* plants were grown in a growth room at 22°C and 75% relative humidity with a 10 h : 14 h, light : dark cycle (short day). *Nicotiana benthamiana* plants were grown at 22°C and 70% relative humidity with a 12 h : 12 h, light : dark cycle.

### Electrophoretic mobility shift assay (EMSA)

The recombinant GST‐ORA59 protein was purified by glutathione‐agarose beads (BD Biosciences, Franklin Lakes, NJ, USA) and eluted by reduced glutathione. DNA probes labeled with or without biotin at the 3′ end were synthesized by Sangon Biotech (Shanghai, China). A chemiluminescent EMSA Kit (Beyotime, Beijing, China) was used for the EMSA assay. The binding reaction contained purified GST‐ORA59, EMSA/Gel‐Shift binding buffer and probes. The GST protein alone was used as a negative control. The detailed procedure for the EMSA assay can be found in the manufacturer’s manual. All the images were taken using a charge‐coupled device (CCD) camera.

### Western blot assays and coimmunoprecipitation (Co‐IP) assays

For Western blot assays, total proteins were extracted with protein extraction buffer (0.25 M Tris‐HCL (pH 6.8), 4% sodium dodecyl sulfate, 0.1% bromophenol blue, 40% glycerol); polyclonal α‐GFP (BBI Life Science, Shanghai, China), α‐HA (Roche), α‐Flag (Sigma‐Aldrich) and the Clarity Western ECL Substrate System (Bio‐Rad) were used to detect protein accumulation.

For Co‐IP assay, the coding sequences of *RPW8.1*, *ACO4* and *ACO4_38‐175_* were amplified from the cDNA synthesized from mRNA extracted from R1Y4 with the primer pairs indicated in Supporting information Table S1, and then the epitope tag hemagglutinin (HA) was added to the C‐terminal of RPW8.1 and the epitope tag Flag to the C‐terminal of ACO4_38‐175_ and ACO4 at the *Bam*HI/*Stu*I sites to make fusion constructs expressing RPW8.1‐HA, ACO4_38‐175_‐Flag and ACO4‐Flag, respectively. Then, RPW8.1‐HA was transiently coexpressed with ACO4_38‐175_‐Flag or ACO4‐Flag in *N. benthamiana* via agroinfiltration. Infiltrated leaves were collected at 36 h postinfiltration (hpi) and were ground in the ice‐cold IP buffer (10% glycerol; 25 mM Tris‐HCl pH 7.5; 1 mM EDTA; 100 mM NaCl; 10 mM dithiothreitol; 1× Protease Inhibitor Cocktail (Roche)) containing 0.5% triton, and centrifuged at 13 000 ***g*** for 10 min at 4°C. Supernatant (20 μl) was taken and used for input control. Another quantity of supernatant was incubated with α‐Flag agarose (Sigma‐Aldrich) at 4°C for 2 h by a vertical mixer. Beads were then collected and washed three times with the IP buffer, and once with 50 mM Tris‐HCl, pH 7.5. Input and immunoprecipitated proteins were analyzed by immunoblotting (IB) using α‐HA (Roche) or α‐Flag (Sigma‐Aldrich). The blots were imaged by the Clarity Western ECL Substrate system (Bio‐Rad).

## Results

### RPW8.1 interacts with ACO4

**Fig. 1:**
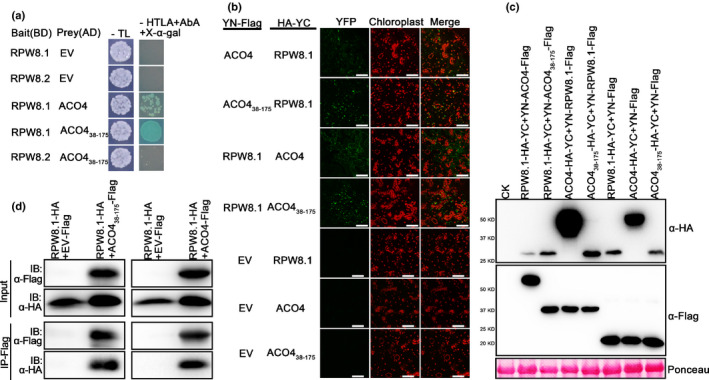
RPW8.1 interacts with 1‐aminocyclopropane‐1‐carboxylate oxidase 4 (ACO4) in yeast and *in planta*. (a) Yeast two‐hybrid (Y2H) assays. *RPW8.1* and *RPW8.2* were translationally fused to the Gal4 DNA‐binding domain (BD) of the pGBKT7 destination vector and the fusion proteins serve as bait. ACO4 and ACO4_38‐175_ were fused to the Gal4 activation domain (AD) of the pGADT7 destination vector and the fusion proteins serve as prey. Cotransformation of empty pGADT7 vector (EV) with pGBKT7 containing RPW8.1 or RPW8.2 was used as negative control. Interactions were indicated by the growth of yeast cells on the selective media SD/–Ade/–His/–Leu/–Trp (‐TLAH) supplemented with X‐α‐gal (40 µg ml^−1^) and Aureobasidin A (200 ng ml^−1^). Plates were photographed after 3 d. (b) Bimolecular fluorescence complementation (BiFC) assays. *RPW8.1*, *ACO4* and *ACO4_38‐175_* were fused to the N‐terminal part (YN) of yellow fluorescent protein (YFP) and/or the C‐terminal part of YFP (YC). YC‐tagged proteins were coexpressed with the YN‐tagged proteins in the *Nicotiana benthamiana* leaves. The pairs of YN empty vector and YC‐tagged RPW8.1, ACO4 or ACO4_38‐175_, were used as negative controls. The YFP fluorescence was determined by confocal microscopy at 2 d after infiltration. From left to right: YFP, chloroplast and merged channels. Bars, 50 μm. (c) Western blot analysis shows the protein expression in (b). Total proteins were extracted and analyzed with the hemagglutinin (HA) and Flag antibody (α‐HA and α‐Flag). Ponceau staining was used as loading control. CK means empty control. (d) *In vivo* coimmunoprecipitation (Co‐IP) assay. HA‐tagged RPW8.1 was coexpressed with Flag‐tagged ACO4_38‐175_ or ACO4 in the *N. benthamiana* leaves. The pair of Flag empty vector (EV‐Flag) and HA‐tagged RPW8.1 was used as a negative control. Total proteins were extracted and subjected to immunoprecipitation of ACO4_38‐175_ or ACO4 by the Flag antibody (α‐Flag), followed by immunoblot analysis with the HA antibody (α‐HA). The input proteins were analyzed with α‐HA and α‐Flag. All the experiments were repeated three times with similar results.

In order to investigate the molecular mechanisms underlying RPW8.1‐mediated immunity in *Arabidopsis*, we performed a Y2H screen (Methods S1) to identify potential interacting proteins of RPW8.1 (as bait). One putative interacting protein was a truncated segment of ACO4 from amino acid residue position 38 to 175 (ACO_38‐175_) (Fig. 1a). The interaction was also detected using the full‐length ACO4 (Fig. 1a). However, we did not detect interaction between RPW8.2 and ACO_38‐175_ in yeast (Fig. 1a), although RPW8.2 shares 45% identity and 65% similarity in amino acid sequences with RPW8.1 (Xiao *et al*., 2001). Thus, we excluded RPW8.2 in the subsequent investigation.

To confirm the interaction between RPW8.1 and ACO4, we performed bimolecular fluorescence complementation (BiFC) (Methods S2) and Co‐IP assays. In the BiFC assay, we detected the reconstituted YFP signal upon transiently coexpressing RPW8.1‐YC with YN‐ACO4_38‐175_ or YN‐ACO4, and YN‐RPW8.1 with ACO4_38‐175_‐YC or ACO4‐YC in *N. benthamiana* (Fig. 1b,c), indicating the interaction between RPW8.1 and ACO4_38‐175_ or ACO4. In the Co‐IP assay, when the ACO4_38‐175_‐Flag or ACO4‐Flag was immunoprecipitated from the protein extracts using Flag antisera, RPW8.1‐HA was detected in the immunocomplex of ACO4_38‐175_‐Flag or ACO4‐Flag by the HA antisera (Fig. 1d), indicating the interaction between RPW8.1 and ACO4_38‐175_ or ACO4. By contrast, no signal was observed in the reactions when RPW8.1‐HA was coexpressed with the Flag empty vector (EV‐Flag) in *N. benthamiana* (Fig. 1d). It has recently been shown that a homolog of RPW8, HR4‐Fei‐0, can form oligomers (Li *et al*., 2020). Thus, we examined whether RPW8.1 interacts with itself by using a Co‐IP assay, with ACO4_38‐175_‐Flag as a positive control. The results showed that RPW8.1 appeared to interact with itself in *N. benthamiana* (Fig. S1). Taken together, these data indicate that RPW8.1 interacts with ACO4_38‐175_, ACO4 and itself.

To explore the specificity of the interaction between ACO4 and RPW8.1, we conducted a series of Y2H assays (Methods S1). First, we divided ACO4 into three fragments based on the sequence signature (Fig. S2a), including ACO4_38‐175_, A4T1 (encoding amino acids (aa) 153–254) and A4T2 (encoding aa 255–323) (Fig. S2a). While we detected interactions in the positive control and between RPW8.1 and ACO4_38‐175_, we did not detect interaction between RPW8.1 and A4T1 or A4T2 (Fig. S2c), indicating that aa 38–175 of ACO4 is essential for the RPW8.1 and ACO4 interaction. Then, we tested whether ACO1 or ACO2 interacts with RPW8.1 because they share high similarity in amino acid sequences with ACO4, and are the closest to ACO4 in the ACO family in *Arabidopsis* (Fig. S2b). We cloned the coding regions of ACO1 and ACO2 into the GAL4 activation domain (AD) vector as the prey proteins and performed Y2H assays. Again, we did not detect interaction between RPW8.1 and ACO1 or ACO2 (Fig. S2d). Therefore, we concluded that the interaction between RPW8.1 and ACO4 is specific and the region from aa 38 to 175 of ACO4 contains the interaction site. Because ACO4 is involved in the ethylene biosynthesis and is responsible for converting ACC to ethylene (Dorling & McManus, 2018), its interaction with RPW8.1 implies that RPW8.1 may influence the ethylene‐signaling pathway.

### Ectopic expression of *RPW8.1* leads to increased ethylene production and signaling

**Fig. 2:**
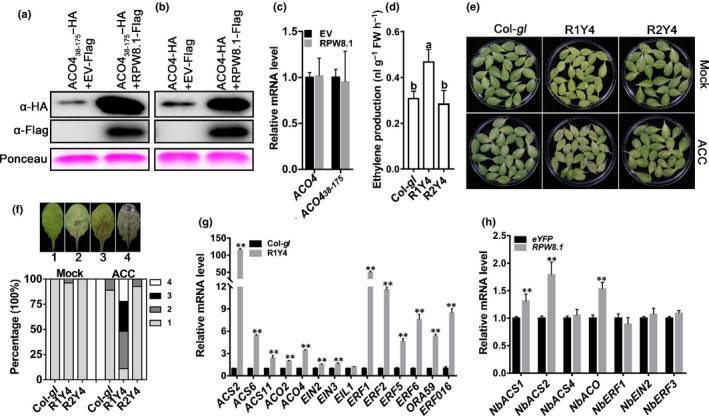
Ectopic expression of *RPW8.1* leads to higher ethylene production and activation of ethylene signaling. (a, b) Western blot (WB) assays. *Agrobacterium* cells harboring *ACO4_38‐175_‐HA* or *ACO4‐HA* were coinfiltrated with or without *RPW8.1‐Flag* in *Nicotiana benthamiana* leaves. Total proteins extracted from *N. benthamiana* leaves were subjected to WB analysis with hemagglutinin (HA) antibody (α‐HA) and Flag antibody (α‐Flag) at 2 d after infiltration. Ponceau staining was used as loading control. (c) Reverse transcription quantitative polymerase chain reaction (RT‐qPCR) assay. *Agrobacterium* cells harboring *ACO4_38‐175_‐HA* or *ACO4‐HA* were coinfiltrated with or without *RPW8.1‐Flag* in *N. benthamiana* leaves. Total RNAs were subjected to RT‐qPCR analysis. *NbEF‐1a* was used as internal control. Error bars indicate SD (*n* = 3). (d) Ethylene biosynthesis rate assay. Seedlings of 2‐wk‐old Col‐*gl*, R1Y4 and R2Y4 were used to measure the ethylene biosynthesis rates. Error bars indicate standard deviation (SD) (*n* = 3). Different letters above the bars indicate significant differences (*P* < 0.01) determined by one‐way ANOVA. (e) The senescence assay. Detached leaves from 5‐wk‐old Col‐*gl*, R1Y4 and R2Y4 plants were treated with water (Mock) or 100 μM 1‐aminocyclopropane‐1‐carboxylic acid (ACC) under dark conditions for 4 d. (f) Quantitative analysis on the senescence leaves in (e). The leaves were classified into four types. Around 60 leaves from plants of each genotype were analyzed. (g) RT‐qPCR assay. The relative expressions of the indicated genes in R1Y4 were calculated relatively to that of Col‐*gl*. *ACT2* was used as an internal control. Error bars indicate SD (*n* = 3). Asterisks (**) above the bars indicate significant differences (*P* < 0.01) determined by Student’s *t*‐test. (h) RT‐qPCR shows the relative expressions of the indicated genes in *N. benthamiana*. *Agrobacterium* strains harboring the Pro_RPW8.1_:*RPW8.1‐eYFP* or Pro_RPW8.1_:*eYFP* were infiltrated into the same *N. benthamiana* leaf side by side, cDNA was synthesized from total RNA extracted from *N. benthamiana* leaves at 2 d after infiltration, and then RT‐qRCR analysis was performed. *NbEF‐1a* was used as internal control. Error bars indicate SD (*n* = 3). Asterisks (**) above the bars indicate significant differences (*P* < 0.01) determined by Student's *t*‐test.

To investigate a possible physiological function of the interaction between RPW8.1 and ACO4, we first tested whether RPW8.1 impacts the protein abundances of ACO4_38‐175_ and ACO4. To this end, we made the constructs expressing ACO4_38‐175_‐HA, ACO4‐HA and RPW8.1‐Flag. RPW8.1‐Flag was transiently coexpressed with ACO4_38‐175_‐HA or ACO4‐HA in *N. benthamiana* leaves via agroinfiltration, and the protein abundances were examined by Western blot. We found that the protein abundances of ACO4_38‐175_‐HA and ACO4‐HA were obviously higher when they were coexpressed with RPW8.1‐Flag than when they were expressed alone (Fig. 2a,b), indicating that RPW8.1 may bind to and stabilize ACO4. We also measured the mRNA expression of *ACO4* and *ACO4_38‐175_* in the infiltrated *N. benthamiana* leaves and found that their expression was not affected by RPW8.1 (Fig. 2c). In a previous study, high‐level expression of *ACO2* and *ACO4* was attributed to increased ethylene release upon cadmium exposure in *Arabidopsis* (Schellingen *et al*., 2014). We thus speculated that RPW8.1 might enhance ethylene production. To further explore the function of RPW8.1 in ethylene biosynthesis, we conducted an ethylene production assay (Methods S3). Because there are five *ACO* genes in *Arabidopsis*, they may be functional redundancy. Therefore, we tested whether loss of *ACO4* impacts ethylene production. As shown in Fig. S3, the loss‐of‐function *aco4* mutant showed lower ethylene production than did Col‐*gl*. Next we measured the ethylene production in R1Y4, Col‐*gl* and R2Y4. As shown in Fig. 2(d), R1Y4 showed higher ethylene production than Col‐*gl* and R2Y4. Consistently with this, the detached leaves of R1Y4 exhibited enhanced dark‐ and ACC‐induced senescence (Methods S4) in comparison with those of Col‐*gl* and R2Y4 (Fig. 2e,f). Taken together, these results indicate that RPW8.1 binds to and stabilizes ACO4 to promote ethylene biosynthesis.

To further validate the role of *RPW8.1* in ethylene biosynthesis and ethylene signaling, we performed a reverse transcription quantitative polymerase chain reaction (RT‐qPCR) assay (Methods S5) to examine the expression of 14 genes (i.e. *ACS2*, *ACS6*, *ACS11*, *ACO2*, *ACO4*, *EIN2*, *EIN3*, *EINL1*, *ERF1*, *ERF2*, *ERF5*, *ERF6*, *ERF016* and *ORA59*) reported to be involved in ethylene biosynthesis and/or ethylene signaling (Peng *et al*., 2008; Chang *et al*., 2013). As shown in Fig. S4, except for *EIN3* and *EIL1*, the remaining 12 genes were indeed induced by ACC treatment. Next, we examined the expression of these genes in R1Y4 and Col‐*gl* and found higher expression of *ACS2*, *ACS6*, *ACS11*, *ACO2*, *ACO4*, *ERF1*, *ERF5*, *ERF6*, *ORA59* and *ERF016* in R1Y4 than in Col‐*gl* (Fig. 2g), strongly suggesting that RPW8.1 may promote ethylene biosynthesis to activate ethylene signaling. To further confirm this conclusion, we transiently expressed *RPW8.1‐e*YFP or *eYFP* in *N. benthamiana*. The RT‐qPCR assays showed that *RPW8.1‐eYFP* induced higher expression of *NbACS1*, *NbACS2* and *NbACO* than the *eYFP* control (Fig. 2h). These results collectively suggest that RPW8.1 promotes ethylene biosynthesis and ethylene signaling via interacting with and stabilizing ACO4.

Next, we examined whether RPW8.1 in R1Y4 induces stronger ethylene‐dependent triple response in seedlings and found no significant difference between R1Y4 and Col‐*gl*, while the ethylene signaling mutants *ein2‐1* and *ein3‐1 eil1‐1* did not show the triple response as expected (data not shown). We speculated that *RPW8.1* expression in very young seedlings might be too low to produce any significant impact on ethylene signaling. Thus, we measured the expression of *RPW8.1* in 1‐wk‐old seedlings and 6‐wk‐old plants of R1Y4 grown on half‐strength MS media and found that the level of *RPW8.1* expression was indeed significantly lower in the younger seedlings than in the 6‐wk‐old plants (Fig. S5).

### *ACO4* plays a negative role in RPW8.1‐mediated immunity

**Fig. 3:**
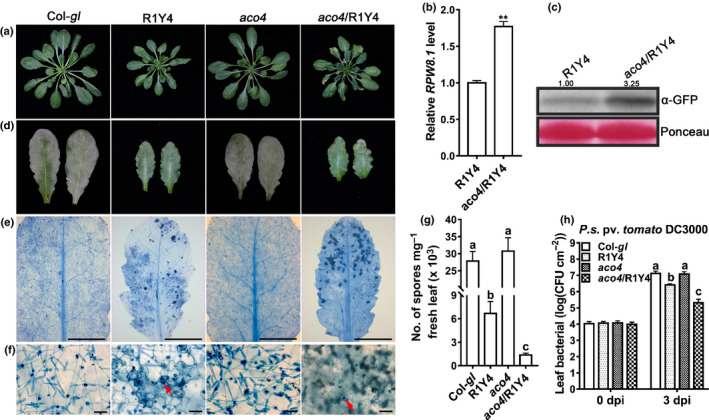
*ACO4* negatively impacts RPW8.1‐mediated cell death and disease resistance. (a) Phenotypes of the indicated lines at 6 wk old. (b) Relative expression levels of *RPW8.1* were measured by reverse transcription quantitative polymerase chain reaction (RT‐qPCR) using *ACT2* as an internal control. Error bars indicate standard deviation (SD) (*n* = 3). Asterisks (**) above the bars indicate significant differences (*P* < 0.01) determined by Student's *t*‐test. (c) Western blot analysis shows the protein abundances of RPW8.1 in R1Y4 and *aco4*/R1Y4. Total proteins were extracted and analyzed with the green fluorescent protein (GFP) antibody (α‐GFP). Ponceau staining was used as loading control. (d) Representative leaves show the disease phenotype of powdery mildew at 10 d postinoculation (dpi) in the indicated lines. (e, f) Representative infected leaves (e) and leaf sections (f) show the fungal infection‐associated cell death and sporulation of powdery mildew stained with Trypan blue at 10 dpi from the indicated lines. The red arrow indicates fungal structures and fungus‐induced cell death. Bars, 5 μm (e); 100 μm (f). (g) Quantification of powdery mildew sporulation on the indicated lines at 10 dpi. Nine infected leaves from each genotype were collected, weighed and subjected to quantitative measurement of spore number mg^–1^ fresh tissue. Error bars indicate SD (*n* = 3). Different letters above the bars indicate significant differences (*P* < 0.01) determined by one‐way ANOVA. (h) Bacterial growth assay for the *Pseudomonas syringae* pv*. tomato* DC3000 (*Pst* DC3000) in the indicated lines. Error bars indicate SD (*n* = 6). Different letters above the bars indicate significant differences (*P* < 0.01), determined by one‐way ANOVA.

To explore the role of ACO4 in RPW8.1‐mediated immunity, we generated the *aco4*/R1Y4 mutant by crossing *aco4* with R1Y4. To our surprise, plants of *aco4*/R1Y4 exhibited obvious spontaneous leaf cell death, whereas no cell death was visible in plants of either R1Y4 or *aco4* at the same age (Figs 3a, S6a,b). Supporting this, the ion leakage level (Methods S3) in *aco4*/R1Y4 was higher compared with that in R1Y4 (Fig. S6d). In addition, H_2_O_2_ accumulation was obviously greater in *aco4*/R1Y4 than in R1Y4, while it was not detectable in Col‐*gl* and *aco4* (Fig. S6c,e). These observations suggest that ACO4 plays a role in repressing RPW8.1‐mediated cell death and H_2_O_2_ production.

Then, we measured the expression of *RPW8.1* and found that *RPW8.1* transcription was elevated in *aco4*/R1Y4 (Fig. 3b). Meanwhile, the RPW8.1 protein abundance in *aco4*/R1Y4 was at least three times higher than that in R1Y4 (Fig. 3c). These results indicate that *ACO4* plays a negative role in the expression of *RPW8.1*.

Next, we inoculated plants of *aco4*/R1Y4, R1Y4, Col‐*gl* and *aco4* with the powdery mildew isolate *G. cichoracearum* UCSC1 (Methods S6). We found that the fungal mass was less abundant in *aco4*/R1Y4 than in R1Y4, and significantly less than that in the susceptible Col‐*gl* and *aco4* (Fig. 3d). To more accurately assess the disease reaction phenotypes, we performed Trypan blue staining to reveal the fungal growth and the fungal infection‐triggered cell death. Microscopic examination revealed that the infected leaves of *aco4*/R1Y4 exhibited more extensive cell death than those of R1Y4, while no cell death was observed in infected leaves of Col‐*gl* and *aco4* (Fig. 3e,f). Quantification of sporulation showed that the total number of spores produced mg^–1^ fresh leaf tissue in *aco4*/R1Y4 was significantly lower than that in R1Y4, indicating enhanced resistance in *aco4*/R1Y4 (Fig. 3g). Then, we wanted to confirm whether *aco4*/R1Y4 has enhanced resistance to other pathogens. We thus tested plants of the four genotypes with *Pseudomonas syringae* pv. *tomato* DC3000 (*Pst* DC3000) (Methods S7), a bacterial strain virulent on Col‐*gl*. We found that *aco4*/R1Y4 supported significantly less bacterial growth than did R1Y4, indicating that *aco4*/R1Y4 also has enhanced resistance to *Pst* DC3000 (Fig. 3h). We also examined the expression of three defense‐related genes, *FRK1* (a marker gene for PTI), *PR1* and *PR2* (marker genes for SA signaling) and found that *aco4*/R1Y4 had higher expression levels for all the three genes than did R1Y4 (Fig. S6f–h). Together, these results indicate that *ACO4* plays a negative role in RPW8.1‐mediated disease resistance.

### The ethylene‐signaling pathway represses RPW8.1‐mediated immunity

**Fig. 4:**
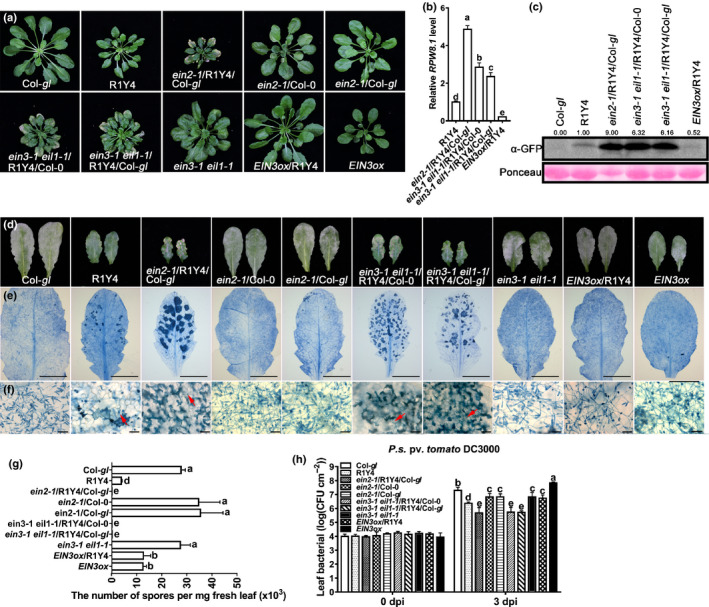
Ethylene signaling attenuates RPW8.1‐mediated cell death and disease resistance. (a) Phenotypes of the indicated lines at 6 wk old. (b) Relative expression levels of *RPW8.1* were measured by reverse transcription quantitative polymerase chain reaction (RT‐qPCR) using *ACT2* as an internal control. Error bars indicate standard deviation (SD) (*n* = 3). Different letters above the bars indicate significant differences (*P* < 0.01) determined by one‐way ANOVA. (c) Western blot analysis shows the protein abundances of RPW8.1 in the indicated lines. Total proteins were extracted and analyzed with the green fluorescent protein (GFP) antibody (α‐GFP). Ponceau staining was used as loading control. (d) Representative leaves show the disease phenotype of powdery mildew at 10 d post‐inoculation (dpi) in the indicated lines. (e, f) Representative infected leaves (e) and leaf sections (f) show the fungal infection‐ associated cell death and sporulation of powdery mildew stained with Trypan blue at 10 dpi from the indicated lines. The red arrow indicates fungal structures and fungus‐induced cell death. Bars, 5 μm (e); 100 μm (f). (g) Quantification of powdery mildew sporulation on the indicated plants at 10 dpi. Nine infected leaves from each genotype were collected, weighed and subjected to quantitative measurement of spore number mg^–1^ fresh tissue. Error bars indicate SD (*n* = 3). Different letters above the bars indicate significant differences (*P* < 0.01) determined by one‐way ANOVA. (h) Bacterial growth assay for the *Pseudomonas syringae* pv.* tomato* DC3000 (*Pst* DC3000) in the indicated lines. Error bars indicate SD (*n* = 6). Different letters above the bars indicate significant differences (*P* < 0.01) determined by one‐way ANOVA.

To assess the role of the ethylene‐signaling pathway in RPW8.1‐mediated immunity more extensively, we introduced the respective *ein2* and *ein3 eil1* mutations and the *EIN3* transgene *EIN3ox* for overexpression into the R1Y4 background by genetic crossing. Phenotypic analysis showed that RPW8.1‐mediated cell death was much more severe in plants of *ein2‐1*/R1Y4/Col‐*gl*, *ein3‐1 eil1‐1*/R1Y4/Col‐0 and *ein3‐1 eil1‐1*/R1Y4/Col‐*gl* than in R1Y4 (Figs 4a, S7a,b). By contrast, the cell death was completely abolished in *EIN3ox*/R1Y4 (Figs 4a, S7a,b). Consistent with this, the rate of ion leakage was higher in *ein2‐1*/R1Y4/Col‐*gl*, *ein3‐1 eil1‐1*/R1Y4/Col‐0 and *ein3‐1 eil1‐1*/R1Y4/Col‐*gl* than in R1Y4 (Fig. S7d), but was reduced in *EIN3ox*/R1Y4 to a value that was comparable to that in Col‐*gl* (Fig. S7d). Moreover, H_2_O_2_ accumulation was obviously greater in *ein2‐1*/R1Y4/Col‐*gl*, *ein3‐1 eil1‐1*/R1Y4/Col‐0 and *ein3‐1 eil1‐1*/R1Y4/Col‐*gl* than in R1Y4, but was not detectable in *EIN3ox*/R1Y4 (Fig. S7c,e). These data indicate that the ethylene‐signaling pathway limits RPW8.1‐mediated cell death and H_2_O_2_ production.

To test if *RPW8.1* expression is repressed by ethylene signaling in general, we examined the expression of *RPW8.1* in the described lines and found that the *RPW8.1* expression was elevated in *ein2‐1*/R1Y4/Col‐*gl*, *ein3‐1 eil1‐1*/R1Y4/Col‐0 and *ein3‐1 eil1‐1*/R1Y4/Col‐*gl* (Fig. 4b) compared with that in R1Y4. The protein abundances of RPW8.1 were also higher in *ein2‐1*/R1Y4/Col‐*gl*, *ein3‐1 eil1‐1*/R1Y4/Col‐0 and *ein3‐1 eil1‐1*/R1Y4/Col‐*gl* than in R1Y4 (Fig. 4c). However, both the mRNA and protein abundances of RPW8.1 were significantly lower in *EIN3ox*/R1Y4 than in R1Y4 (Fig. 4b,c). These results indicate that the ethylene‐signaling pathway represses the expression of *RPW8.1*.

Next, we examined the disease reaction phenotypes of the various lines with *G. cichoracearum* UCSC1 and *Pst* DC3000 (Methods S6 and S7). As expected, the results showed that the fungal mass was less abundant in *ein2‐1*/R1Y4/Col‐*gl*, *ein3‐1 eil1‐1*/R1Y4/Col‐0 and *ein3‐1 eil1‐1*/R1Y4/Col‐*gl*, but more abundant in *EIN3ox*/R1Y4 than in R1Y4 (Fig. 4d). Consistently, the fungal infection‐induced cell death was obviously more severe in *ein2‐1*/R1Y4/Col‐*gl*, *ein3‐1 eil1‐1*/R1Y4/Col‐0 and *ein3‐1 eil1‐1*/R1Y4/Col‐*gl*, but less severe in *EIN3ox*/R1Y4 than in R1Y4 (Fig. 4e,f). The total number of spores mg^–1^ leaf tissue was significantly lower in *ein2‐1*/R1Y4/Col‐*gl*, *ein3‐1 eil1‐1*/R1Y4/Col‐0 and *ein3‐1 eil1‐1*/R1Y4/Col‐*gl*, but higher in *EIN3ox*/R1Y4 than in R1Y4 (Fig. 4g), indicating enhanced resistance in *ein2‐1*/R1Y4/Col‐*gl*, *ein3‐1 eil1‐1*/R1Y4/Col‐0, *ein3‐1 eil1‐1*/R1Y4/Col‐*gl,* but compromised resistance in *EIN3ox*/R1Y4. Moreover, the bacterial strain *Pst* DC3000 showed significantly less growth in *ein2‐1*/R1Y4/Col‐*gl*, *ein3‐1 eil1‐1*/R1Y4/Col‐0 and *ein3‐1 eil1‐1*/R1Y4/Col‐*gl*, but more in *EIN3ox*/R1Y4 than in R1Y4 (Fig. 4h). Consistent with the disease phenotypes, the expression levels of *FRK1*, *PR1* and *PR2* were significantly higher in *ein2‐1*/R1Y4/Col‐*gl*, *ein3‐1 eil1‐1*/R1Y4/Col‐0 and *ein3‐1 eil1‐1*/R1Y4/Col‐*gl*, but lower in *EIN3ox*/R1Y4 compared with R1Y4 (Fig. S7f–h). These results indicate that the ethylene‐signaling pathway plays a negative role in RPW8.1‐mediated disease resistance.

### ORA59, ERF6 and ERF016 trans‐repress the activity of the *RPW8.1* promoter

**Fig. 5:**
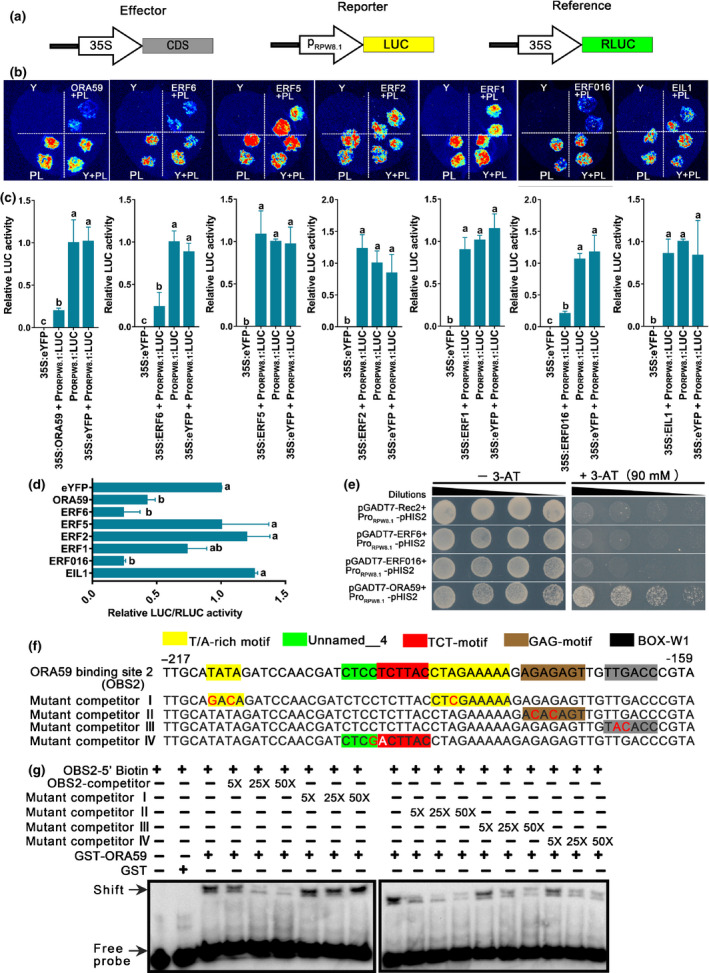
Ethylene‐responsive factors EFR6, ERF016 and ORA59 trans‐repress the *RPW8.1* promoter and ORA59 binds to it. (a) Schematic representation of the constructs used in the luciferase (LUC) assay or the dual‐luciferase reporter assay. CDS, different genes; RLUC, renilla luciferase. (b) LUC images of *Nicotiana benthamiana* leaves coinfiltrated with the indicated combinations of the constructs. The *eYFP* was used as a control. LUC activity was detected using a low‐light cooled charge‐coupled device imaging apparatus at 2 d after infiltration. Y, eYFP; L, LUC; PL, P_RPW8.1_‐LUC. (c) Quantitative analysis of the LUC intensity in (b) using imagej. Four independent leaves were assessed. Error bars indicate standard deviation (SD) (*n* = 4). Different letters above the bars indicate significant differences (*P* < 0.01) determined by one‐way ANOVA. (d) Dual‐luciferase reporter assay in *N. benthamiana*. The LUC and RLUC activities at the site of infiltration were measured. The ratio LUC : RLUC of the eYFP plus the *RPW8.1* promoter was considered as a calibrator (set to 1). Error bars indicate SD (*n* = 3). Different letters above the bars indicate significant differences (*P* < 0.01) determined by one‐way ANOVA. (e) Yeast one‐hybrid (Y1H) assay shows that ORA59, but not ERF6 and ERF016, binds to the full length of the *RPW8.1* promoter. The full length of the *RPW8.1* promoter was fused to the pHIS2 vector. *ERF6*, *ERF016* and *ORA59* were fused to the pGADT7 vector. Yeast cells grown on the selective medium SD/‐Trp/‐Leu/‐His supplemented with 90 mM 3‐amino‐1,2,4‐triazole (3‐AT) indicated the interactions of the indicated combinations of the constructs. The pair of pGADT7‐Rec2 together with Pro_RPW8.1_‐pHIS2 were used as a negative control. Plates were photographed after 3 d. (f) Sequences of ORA59 binding site 2 (OBS2). Different mutant oligonucleotides act as the mutant competitors of biotinylated OBS2 probe (i.e. mutant competitor I is a mutant of T/A‐rich motif; mutant competitor II is a mutant of GAG‐motif; mutant competitor III is a mutant of BOX‐W1; mutant competitor IV is a mutant of unnamed‐motif and TCT‐motif). Motifs in the OBS2 are highlighted with different colors. (g) Electrophoretic mobility shift assay. OBS2‐competitor and the different mutant oligonucleotides were used for competitive binding in five‐, 25‐ and 50‐fold excess of the biotinylated probe.

To understand how the ethylene‐signaling pathway attenuates RPW8.1‐mediated immunity, we arbitrarily selected seven representative TFs involved in the ethylene‐signaling pathway to test if any of them can suppress the activity of the *RPW8.1* promoter via the luciferase (LUC) reporter assay (Methods S8). To this end, the full‐length *RPW8.1* promoter was fused to LUC (P_RPW8.1_‐LUC) as the reporter, and the *TF* genes were expressed from the CaMV *35S* promoter as the effectors (Fig. 5a). We found that the expression of P_RPW8.1_‐LUC was significantly suppressed by ORA59, ERF6 or ERF016, but not by ERF5, ERF2, ERF1 or EIL1 (Fig. 5b,c). To assess the trans‐suppression of the *RPW8.1* promoter activity by the selected TFs more accurately, we performed a dual‐luciferase reporter assay in which Renilla luciferase (RLUC) was used as an internal control. Consistent with the earlier results, we found that ORA59, ERF6 and ERF016 significantly suppressed the activity of the *RPW8.1* promoter (Fig. 5d).

### ORA59 trans‐represses the activity of the *RPW8.1* promoter via direct binding to a T/A‐rich motif

The AP2/ERF domain of ERFs is known to bind specifically to GCC (AGCCGCC) and/or DRE/CRT (A/GCCGAC) boxes (Hao *et al*., 1998). Such boxes were not detected in the *RPW8.1* promoter using the online tool at http://bioinformatics.psb.ugent.be/webtools/plantcare/html/. However, the AP2/ERF domain is predicted to bind to the *RPW8.1* promoter when analyzed with the online tool at http://plantpan2.itps.ncku.edu.tw/promoter.php. To test this prediction, we performed a Y1H assay (Methods S9) to evaluate whether ORA59, ERF6 or ERF016 binds to the *RPW8.1* promoter. The result showed that ORA59, not ERF6 or ERF016, directly interacted with the *RPW8.1* promoter (Fig. 5e). To identify the interaction site in the *RPW8.1* promoter, we divided the *RPW8.1* promoter into five fragments and subjected them to Y1H assays. As shown in Fig. S8, all the yeast cells transformed with the indicated combination of the constructs could grow on the SD/‐Trp/‐Leu/‐His medium without 3‐AT. However, in the presence of 90 mM 3‐AT, only the cells cotransformed with pGADT7‐ORA59 and Pro_RPW8.1_‐pHIS2‐III, and pGADT7‐ORA59 and Pro_RPW8.1_‐pHIS2‐V grew well (Fig. S8d,f), indicating that ORA59 binds directly to fragment III and fragment V of the *RPW8.1* promoter. The cells cotransformed with pGADT7‐Rec2 and Pro_RPW8.1_‐pHIS2‐II, or pGADT7‐Rec2 and Pro_RPW8.1_‐pHIS2‐IV also grew, indicating that Pro_RPW8.1_‐pHIS2‐II and Pro_RPW8.1_‐pHIS2‐IV exhibited self‐activation (Fig. S8c,e). However, the cells cotransformed with pGADT7‐ORA59 and Pro_RPW8.1_‐pHIS2‐I showed no growth (Fig. S8b). As expected, the cells cotransformed with pGADT7‐ERF6 (or pGADT7‐ERF016) plus Pro_RPW8.1_‐pHIS2‐I or Pro_RPW8.1_‐pHIS2‐III or Pro_RPW8.1_‐pHIS2‐V did not show any growth (Fig. S8). These results suggest that ORA59 may bind to the *RPW8.1* promoter via the region from −707 to −476 bp and −277 to −1 bp upstream of the ATG start codon. Because the region from −277 to −1 bp is close to start codon, we prioritized our efforts on studying this fragment.

To confirm the binding activity and identify the binding site of ORA59, we further divided the fragment V (−277 to −1 bp) into five parts, termed ORA59 binding site 1 (OBS1) to OBS5 (Fig. S9a). We then synthesized them as probes with biotin‐labeling and deployed them in the EMSA (Fig. S9b) (Methods S10). The EMSA assay showed that GST‐ORA59 could bind all five probes (Fig. S9c). We analyzed the core elements in these five fragments and identified an overrepresented DNA motif (named T/A‐rich motif) that may be the binding site of GST‐ORA59 (Fig. S9b). To confirm this, we synthesized probes separately with mutations in the different motifs to be the competitors of OBS2, including T/A‐rich‐, TCT‐, GAG‐, BOX‐W1 and unnamed motifs (Fig. 5f). Indeed, the GST‐ORA59 binding signal did not change with the increase of the mutant competitor I, which is a mutant of T/A‐rich motif (Fig. 5g). In addition, we synthesized another T/A‐rich motif mutant competitor V of OBS1 (Fig. S9b). Again, the ORA59 binding signal did not change with the increase of the mutant competitor V (Fig. S9d), while the binding signal was obviously reduced with the increase of the OBS1‐competitor (Fig. S9d), OBS2‐competitor, mutant competitor II, mutant competitor III or mutant competitor IV (Fig. 5g). Taken together, these results indicate that ORA59 binds directly to the *RPW8.1* promoter via a T/A‐rich motif(s).

### *ERF6*, *ERF016* and *ORA59* negatively regulate RPW8.1‐mediated immunity

**Fig. 6:**
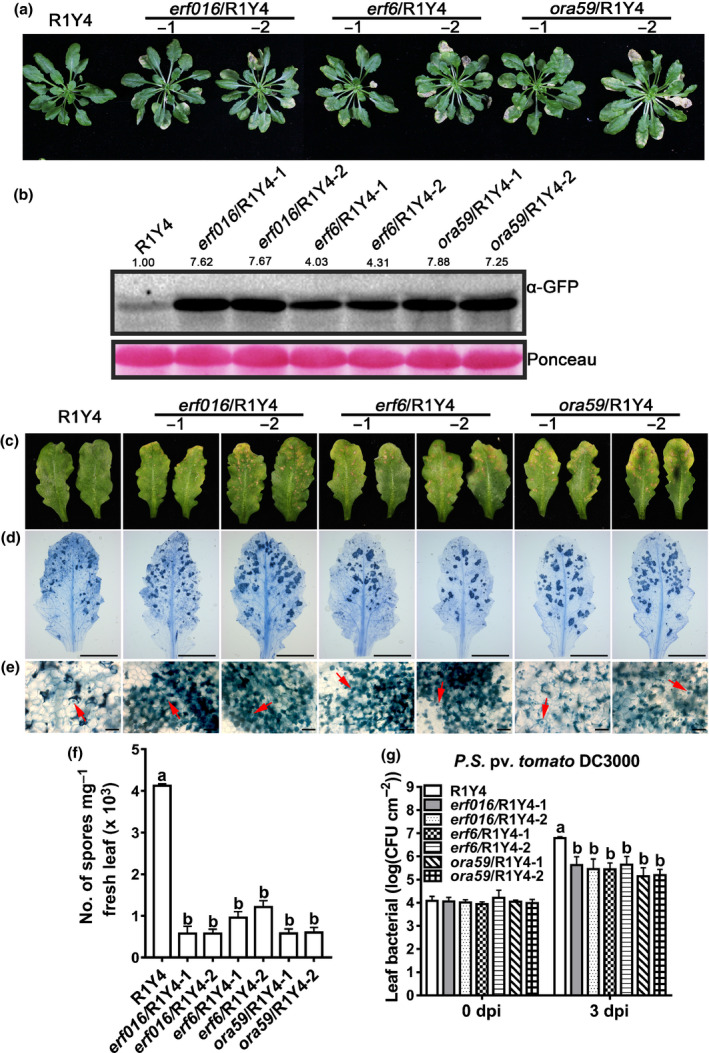
Ethylene‐responsive factors (ERFs) negatively regulate RPW8.1‐mediated cell death and disease resistance. (a) Phenotypes of the indicated mutants in the R1Y4 background at 6 wk old. (b) Western blot assay shows the protein abundances of RPW8.1 in the indicated lines. Total proteins were extracted and analyzed with the α‐green fluorescent protein antibody (α‐GFP). Ponceau staining was used as loading control. (c) Representative leaves show the disease phenotype of powdery mildew at 10 d post‐inoculation (dpi) in the indicated lines. (d, e) Representative infected leaves (d) and leaf sections (e) show the fungal infection‐associated cell death and sporulation of powdery mildew stained with Trypan blue at 10 dpi from the indicated lines. The red arrows indicate fungal structures and fungus‐induced cell death. Bars, 5 μm (d); 100 μm (e). (f) Quantification of powdery mildew sporulation on the indicated plants at 10 dpi. Nine infected leaves from each genotype were collected, weighed and subjected to quantitative measurement of spore number mg^–1^ fresh tissue. Error bars indicate standard deviation (SD) (*n* = 3). Different letters above the bars indicate significant differences (*P* < 0.01) determined by one‐way ANOVA. (g) Bacterial growth assay for the *Pseudomonas syringae* pv.* tomato* DC3000 (*Pst* DC3000) in the indicated plants. Error bars indicate SD (*n* = 6). Different letters above the bars indicate significant differences (*P* < 0.01) determined by one‐way ANOVA.

Because ERF6, ERF016 and ORA59 are capable of reducing *RPW8.1*’s promoter activity (Fig. 5), we reasoned that these TFs probably negatively regulate RPW8.1‐mediated immunity. To validate this inference, we first checked whether expression of *ERF6*, *ERF016* and *ORA59* changes in response to pathogen infection and found that *ORA59* was significantly induced to higher levels upon powdery mildew infection, whereas *ERF6* and *ERF016* were slightly downregulated at some time points after inoculation (Fig. S10). We then deployed the CRISPR/Cas9 technology (Methods S11) to knock out *ERF6*, *ERF016* or *ORA59* in the R1Y4 background and generated two independent homozygous loss‐of‐function mutations (small deletions or insertions) for each gene (Fig. S11b). As expected, we found that RPW8.1‐mediated cell death was more severe in *erf016*/R1Y4‐1, *erf016*/R1Y4‐2, *erf6*/R1Y4‐1, *erf6*/R1Y4‐2, *ora59*/R1Y4‐1 and *ora59*/R1Y4‐2 than in R1Y4 (Figs 6a, S12a,b). Consistent with this observation, the rate of ion leakage was higher in these mutants than in R1Y4 (Fig. S12d). H_2_O_2_ accumulation was also obviously greater in these mutants than in R1Y4 (Fig. S12c,e). Furthermore, the protein abundances of RPW8.1 were higher in these mutants than in R1Y4 (Fig. 6b). Together, these results indicate that *ERF6*, *ERF016* and *ORA59* negatively regulate *RPW8.1* expression and RPW8.1‐mediated H_2_O_2_ production and cell death.

Next, we tested the mutant lines with *G. cichoracearum* UCSC1 and *Pst* DC3000 and found that the fungal growth was further reduced in each of these mutant lines compared with R1Y4, which was associated with enhanced cell death in these mutant lines compared with that in R1Y4 (Fig. 6c–e). Consistent with the disease reaction phenotypes, fungal sporulation was significantly lower in these mutants than in R1Y4 (Fig. 6f). Furthermore, these mutant lines supported less proliferation of the bacterial pathogen *Pst* DC3000 than did R1Y4 (Fig. 6g). In addition, levels of constitutive expression of *FRK*, *PR1* and *PR2* were significantly higher in these mutant lines than those in R1Y4 (Fig. S12f–h). Together, these results indicate that *ERF6*, *ERF016* and *ORA59* negatively regulate RPW8.1‐mediated disease resistance.

To further confirm that the enhanced resistance seen in R1Y4 mutant lines containing mutations in *ERF6*, *ERF016* or *ORA59* is caused by enhanced expression of *RPW8.1*, we also obtained two independent homozygous mutants for each gene in the Col‐*gl* background (Fig. S11c). As expected, all the mutant lines were similar to Col‐*gl* (Fig. S13a,b), showed no spontaneous cell death and H_2_O_2_ production (Fig. S13c,d), and were as susceptible as Col‐*gl* to powdery mildew (Fig. S13e,f).

### Coordinated regulation between *RPW8.1* and *ORA59*

**Fig. 7:**
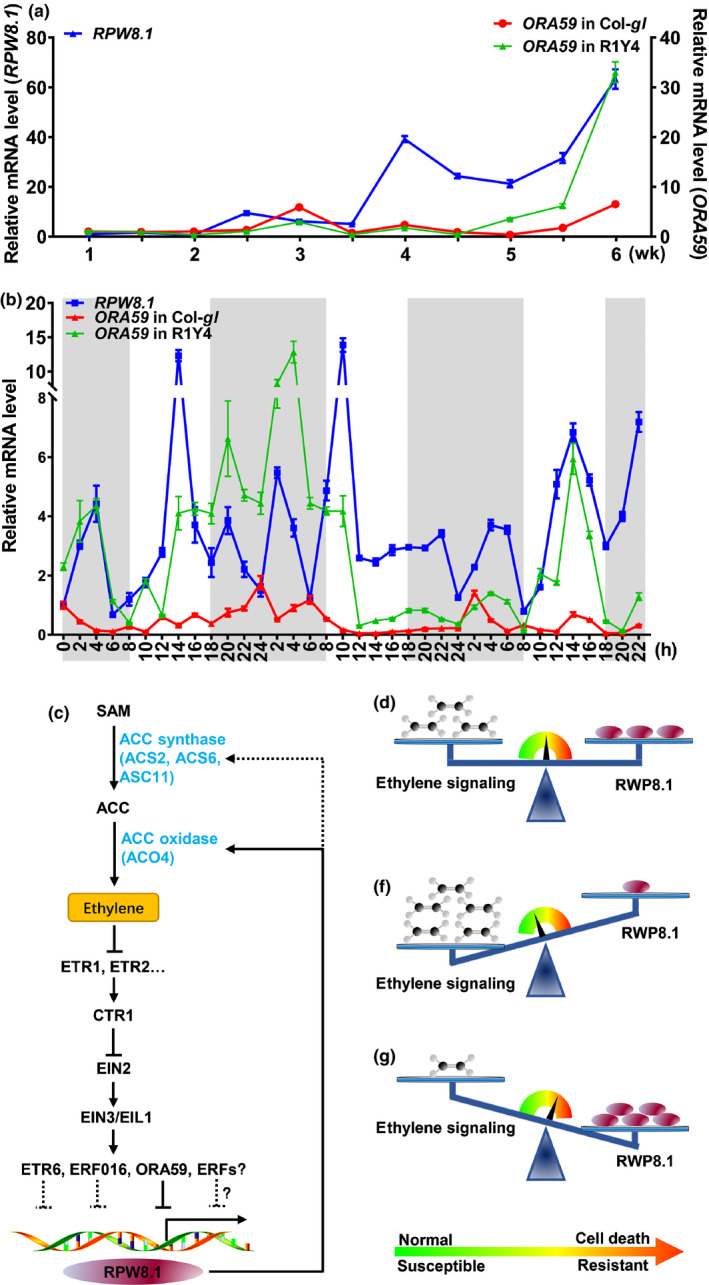
Expression patterns of *ORA59* and *RPW8.1* in Col‐*gl* and R1Y4, and a model illustrating the *RPW8.1* expression – ethylene‐signaling feedback‐regulatory circuit. (a) Reverse transcription quantitative polymerase chain reaction (RT‐qPCR) analysis shows the relative expression of *ORA59* and *RPW8.1* in R1Y4 and Col*‐gl* over the growing season. Samples were collected at the indicated times (wk). *ACT2* was used as an internal control. Error bars indicate standard deviation (SD) (*n* = 3). (b) RT‐qPCR analysis shows the rhythm of *ORA59* and *RPW8.1* expression in R1Y4 and Col*‐gl* at 6 wk old. Samples were collected at the indicated times. *ACT2* was used as an internal control. Gray boxes indicate the dark period. Error bars indicate SD (*n* = 3). (c) Model illustrating the relationship between *RPW8.1* expression and ethylene signaling in *Arabidopsis*. RPW8.1 promotes ethylene production via upregulation of *ACS2*, *ACS6* and *ACS11* transcription, and binding to and stabilizing ACO4. However, ERF6, ERF016 and ORA59 trans‐repress the activity of the *RPW8.1* promoter to attenuate *RPW8.1* expression, thus negatively regulating RPW8.1‐mediated cell death and disease resistance. (d) There may exist a dynamic balance between *RPW8.1* expression and ethylene signaling during the whole plant growth period. (e) Enhanced ethylene signaling disrupts the dynamic balance between *RPW8.1* expression and ethylene signaling. *RPW8.1* expression is attenuated to avoid unnecessary defense responses such as cell death and H_2_O_2_ accumulation. (f) Upon pathogen detection, salicylic acid (SA) signaling is strengthened, which suppresses ethylene signaling, resulting in derepression of *RPW8.1* expression and enhanced cell death and disease resistance.

To understand the negative feedback regulation of *RPW8.1* by *ORA59*, we conducted two time‐course RT‐qPCR assays on the expression of *RPW8.1* and *ORA59* in R1Y4 and Col‐*gl*. First, we examined the expression patterns of *RPW8.1* and *ORA59* over the growing season. As showed in Fig. 7(a), the expression of *RPW8.1* was significantly increased in R1Y4 at 3 wk after planting, while the expression of ORA59 was also increased at 3 wk after planting, especially after 4 wk. However, the expression of ORA59 in Col‐*gl* only increased slightly at 5 wk after planting. Next, we examined the rhythms of *RPW8.1* and *ORA59* expression. As shown in Fig. 7(b), *ORA59* showed an expression pattern similar to that of *RPW8.1* in R1Y4, which is different from the expression pattern of *ORA59* in Col‐*gl*. Together, these results indicate that there is a coordinated regulation between *RPW8.1* and *ORA59*.

### Coordinated regulation between *RPW8.1* and ethylene signaling

To further confirm that RPW8.1 indeed promotes ethylene signaling, which in turn attenuates *RPW8.1* expression and function, we measured *RPW8.1* expression upon ACC treatment in R1Y4, *ein2‐1*/R1Y4/Col‐*gl*, *ein3‐1 eil1‐1*/R1Y4/Col‐0, *ein3‐1 eil1‐1*/R1Y4/Col‐*gl*, *ora59*/ R1Y4‐1, *erf6*/ R1Y4‐1 and *erf016*/ R1Y4‐1. As shown in Fig. S14, upon ACC treatment the expression of *RPW8.1* was significantly reduced in R1Y4, but not in *ein2‐1*/R1Y4/Col‐*gl*, *ein3‐1 eil1‐1*/R1Y4/Col‐0, and *ein3‐1 eil1‐1*/R1Y4/Col‐*gl*. Interestingly, the expression of *RPW8.1* was also significantly reduced upon ACC treatment in *ora59*/ R1Y4‐1, *erf6*/ R1Y4‐1 and *erf016*/ R1Y4‐1, albeit to a lesser extent when compared with that in R1Y4 (Fig. S14). This result suggests that ERF6, ERF016 and ORA59 may act redundantly to repress of *RPW8.1* expression.

## Discussion

Conceivably, the timing and amplitude of plant immune responses are tightly controlled to enable cost‐effective resistance against pathogens. In other words, appropriate downregulation of immune signaling is also important as it can prevent excessive or overly prolonged activation of immune responses in plants. Previously, we found that ectopic expression of *RPW8.1* activates the resistance to powdery mildew and oomycete (Ma *et al*., 2014), and boosts PTI basal defense signaling to activate defense response against virulent pathogens (Li *et al*., 2018). In this study, we provided several lines of evidence to show that ectopic expression of *RPW8.1* also leads to increased ethylene production and ethylene signaling, which, in turn, feedback‐regulates RPW8.1*‐*mediated immunity.

First, we showed that RPW8.1 interacted directly with ACO4, one of the ACC oxidases converting ACC into ethylene (Fig. 1). Such interaction seemed to stabilize ACO4 and promote ethylene production, leading to activation of the ethylene‐signaling pathway (Fig. 2). Second, RPW8.1‐mediated immunity was further enhanced by loss‐of‐function mutations in *ACO4* or in *EIN2*, *EIN3* and *EIL1* encoding key components of the ethylene‐signaling pathway (Figs 3, 4). Conversely, RPW8.1‐mediated immunity was compromised by overexpression of *EIN3* (Fig. 4). Third, the *RPW8.1* promoter activity could be repressed by three ERFs, ERF6, ERF016 and ORA59, of which ORA59 was able bind to the *RPW8.1* promoter via a T/A‐rich motif(s) (Figs 5, S9). Consistent with these results, knocking out each one of the three *ERF*s led to increased *RPW8.1* expression and enhanced immunity (Fig. 6). Lastly, we showed that the expression of *RPW8.1* and *ORA59* exhibited coordinated expression patterns (Fig. 7a,b), which probably reflects a complex positive (*RPW8.1* on *ORA59*) and negative (*ORA59* on *RPW8.1*) transcriptional regulation between these two genes. This offers an explanation of our earlier observation that when *RPW8.1* is expressed above a threshold level as R1Y4 plants grow older (4 wk or more), spontaneous HR‐like cell death becomes visible but then confined (Ma *et al*., 2014). Thus, collectively, our results have revealed a mechanism by which enhanced ethylene signaling is deployed to put a brake on *RPW8.1* expression, thereby fine‐tuning RPW8.1‐mediated immunity.

It would be interesting to know how RPW8.1 stabilizes ACO4, enhances ethylene production and activates the ethylene‐signaling pathway. Upregulation and/or stabilization of the key enzymes, such as ACSs and ACOs, in the ethylene biosynthesis pathway appears to be a common mechanism to enhance ethylene signaling under distinct physiological contexts. For example, in response to flg22 treatment, ACS2 and ACS6 are stabilized upon phosphorylation by MPK6, leading to elevated ethylene production and signaling (Liu & Zhang, 2004). The expression of *ACS2*, *ACS6* and *ACS11* is upregulated by wounding or application of indole‐3‐acetic acid (IAA), resulting in the activation of ethylene signaling (Tsuchisaka & Theologis, 2004). Moreover, *ACS2* and *ACS6*, together with *ACO2* and *ACO4*, are also upregulated during abiotic stress (Schellingen *et al*., 2014). Given these precedents, it may not seem surprising that ACO4 (and perhaps other members such as ACS2, ASC6 and ACS11; Fig. 2g) is expressed at higher levels and gets stabilized to promote ethylene signaling as RPW8.1 accumulates to activate defense. However, it is intriguing that RPW8.1, as an atypical R protein containing a CC domain (Xiao *et al*., 2001), physically interacts with and stabilizes ACO4 which contains two conservative domains (i.e. PcbC (isopenicillin N synthase) and 2OG‐FeII_Oxy (Fig. S2a)). Enzymes with a 2OG‐FeII_Oxy domain typically catalyze the oxidation of an organic substrate (Aravind & Koonin, 2001). The N‐terminal of PcbC domain is highly conserved in the proteins with 2OG‐FeII_Oxy‐dependent dioxygenase activity (Hagel & Facchini, 2010). We found that the region covering aa 38–175 of ACO4 is essential for interacting with RPW8.1 (Fig. S2c). This region belongs to the N‐terminal portion of the PcbC domain that is not the catalytic center but may be important for protein stabilization. However, it remains to be determined whether the binding of RPW8.1 to the PcbC domain is required for stabilization of ACO4.

Results of this study also suggest that ethylene signaling may play dual opposing roles in RPW8.1‐mediated immunity. On the one hand, ethylene signaling may contribute to *RPW8.1*‐mediated basal defense because ethylene signaling appears to synergistically interact with PTI signaling, and RPW8.1 expression can boost ethylene production and PTI signaling; the latter may be partially attributable to RPW8.1‐mediated resistance to pathogens (Li *et al*., 2018). Specifically, the expression of the PAMP receptor gene *FLS2* is positively regulated by EIN3 and EIL1 but impaired as a result of loss of *EIN2* (Boutrot *et al*., 2010). Not surprisingly, the induced expression of *FRK1* as a reporter of PTI upon flg22 treatment is compromised in the *ein2* mutant (Asai *et al*., 2002; Boutrot *et al*., 2010). Interestingly, as in the case of RPW8.1‐mediated defense, PTI signaling also leads to enhanced ethylene production and signaling. For example, while MPK3 and MPK6, two key PTI signaling components, can phosphorylate and thereby stabilize ACS2 and ACS6 to promote ethylene production and signaling, WRKY33, a TF activated during PTI, can bind to the W‐boxes in the promoter of *ACS2* and *ACS6*, thereby also upregulating ethylene biosynthesis (Li *et al*., 2012). On the other hand, contrary to the positive impact of ethylene signaling on PTI, we have generated multiple lines of evidence in this study to demonstrate that elevated ethylene signaling can restrict the expression of *RPW8.1* to attenuate RPW8.1‐mediated cell death and other defense responses (Figs 5, 6). Thus, our results imply that RPW8.1 may possess two functional properties: activation of basal defense (which may be partly or mostly via PTI connection) and activation of stronger, SA‐dependent and cell death‐associated defense. The former form may be strengthened by its connection to PTI and ethylene signaling, whereas the latter appears to be attenuated by ethylene signaling via a feedback circuit. This hypothesis is also compatible with an early observation that the *ein2* mutant is slightly more susceptible to powdery mildew (Xiao *et al*., 2005). However, it should be pointed out that EIN3 has been shown to bind directly to the promoter of *SID2* to inhibit its expression, thus downregulating the biosynthesis of SA and SA‐dependent defense response (Chen *et al*., 2009). Hence, it is also likely that *RPW8.1* expression and its mediated resistance is a result, in part, of the inhibition of the expression of *SID2* through EIN3. Future studies are required to dissect the two inhibitory mechanisms by which ethylene signaling attenuates *RPW8.1* expression and RPW8‐mediated cell death and immunity. In addition, as ethylene signaling, together with JA signaling, is required for resistance against necrotrophic pathogens (Ramirez‐Prado *et al*., 2018), the RPW8.1‐ethylene‐signaling feedback regulation may also play a role in balancing immunity against both necrotrophic and biotrophic pathogens. Lastly, our findings in this study may provide fresh insights into how RPW8.1 and RPW8.2 coordinately activate broad‐spectrum resistance. That is, while *RPW8.2* is strongly induced by SA signaling in epidermal cells to activate anti‐haustorium defense at the EHM, *RPW8.1* is constitutively expressed in mesophyll cells to maintain a basal level of SA‐dependent signaling to boost RPW8.2’s function as well as PTI; and *RPW8.1* itself is under feedback attenuation via its enhanced ethylene signaling to avoid overactivation of defenses. Recognizing the latter properties of RPW8.1 may also help us to better utilize RPW8.1‐mediated broad‐spectrum resistance, through expression of *RPW8.1* within an optimum range and/or selection of plant genotypes in which ethylene signaling is relatively less active.

In conclusion, we have identified a feedback‐regulatory circuit where *RPW8.1* enhances the ethylene‐signaling pathway, which in turn attenuates *RPW8.1* expression (Fig. 7c). This regulatory mechanism may help plants to achieve a dynamic balance between *RPW8.1* expression and ethylene signaling (Fig. 7d). When *RPW8.1* expression is too high, enhanced ethylene signaling puts a brake on it to avoid RPW8.1 overexpression‐triggered autoimmunity (Fig. 7e). Conversely, pathogen‐induced SA signaling suppresses ethylene signaling, thus derepressing *RPW8.1* expression, leading to enhanced defense responses (Fig. 7f).

## Author contributions

Z‐XZ, QF and P‐QL performed most of the experiments with support from X‐RH, J‐HZ, Y‐JX, L‐LZ, Y‐YH, J‐QZ, JF, YL, SX and W‐MW. W‐MW and SX conceived the project. Z‐XZ and W‐MW designed the experiments. Z‐XZ, W‐MW and SX analyzed the data and wrote the manuscript. Z‐XZ and QF contributed equally to this work.

## Supporting information


**Fig. S1** RPW8.1 interacts with itself.
**Fig. S2** RPW8.1 specifically interacts with aa 38–175 of ACO4 in yeast.
**Fig. S3** The *aco4* mutant is compromised in ethylene production.
**Fig. S4** Expression patterns of ethylene‐related genes upon ACC treatment.
**Fig. S5** Expression levels of *RPW8.1* in R1Y4 at two different developmental stages.
**Fig. S6** *ACO4* negatively impacts RPW8.1‐mediated cell death and defense responses.
**Fig. S7** Ethylene signaling plays a negative role in RPW8.1‐mediated cell death and defense responses.
**Fig. S8** ORA59 binds to the truncated fragments of the *RPW8.1* promoter in yeast.
**Fig. S9** ORA59 binds directly to the *RPW8.1* promoter.
**Fig. S10** Transcriptional changes of *ERF6*, *ERF016* and *ORA59* in response to powdery mildew infection.
**Fig. S11** Mutation sites of *ERF016*, *ERF6* and *ORA59* in R1Y4 and Col‐*gl*.
**Fig. S12** *ERF016*, *ERF6* and *ORA59* negatively regulate RPW8.1‐mediated cell death and defense responses.
**Fig. S13** Phenotypic analysis of *ERF6*, *ERF016* and *ORA59* knockout mutants in Col‐*gl*.
**Fig. S14** The expression pattern of *RPW8.1* upon ACC treatment.
**Methods S1** Yeast two‐hybrid (Y2H) assays.
**Methods S2** Bimolecular ﬂuorescence complementation (BiFC) assay.
**Methods S3** Determination of ethylene biosynthesis rates and electrolyte leakage measurements.
**Methods S4** Leaf senescence assay.
**Methods S5** RNA extraction and reverse transcription quantitative PCR (RT‐qPCR).
**Methods S6** Pathogen inoculation and microscopy and analysis.
**Methods S7** Bacterial growth assays.
**Methods S8** Luciferase (LUC) reporter assays in *Nicotiana benthamiana*.
**Methods S9** Yeast one‐hybrid (Y1H) assays.
**Methods S10** Protein expression and purification.
**Methods S11** CRISPR/Cas9 plasmids construction and mutant screening.
**Table S1** Primers and probes used in this study.
Please note: Wiley Blackwell are not responsible for the content or functionality of any Supporting Information supplied by the authors. Any queries (other than missing material) should be directed to the *New Phytologist* Central Office.
