# Supplementary material for: RPW8.1 enhances the ethylene‐signaling pathway to feedback‐attenuate its mediated cell death and disease resistance in Arabidopsis
Source: New Phytol. 2020 Sep 5;229(1):516–31. doi: 10.1111/nph.16857 (PMC7754472; doi:10.1111/nph.16857)
Supplement: Supplementary file 1 — Fig. S1 RPW8.1 interacts with itself. Fig. S2 RPW8.1 specifically interacts with aa 38–175 of ACO4 in yeast. Fig. S3 The aco4 mutant is compromised in ethylene production. Fig. S4 Expression patterns of ethylene‐related genes upon ACC treatment. Fig. S5 Expression levels of RPW8.1 in R1Y4 at two different developmental stages. Fig. S6 ACO4 negatively impacts RPW8.1‐mediated cell death and defense responses. Fig. S7 Ethylene signaling plays a negative role in RPW8.1‐mediated cell death and defense responses. Fig. S8 ORA59 binds to the truncated fragments of the RPW8.1 promoter in yeast. Fig. S9 ORA59 binds directly to the RPW8.1 promoter. Fig. S10 Transcriptional changes of ERF6, ERF016 and ORA59 in response to powdery mildew infection. Fig. S11 Mutation sites of ERF016, ERF6 and ORA59 in R1Y4 and Col‐gl. Fig. S12 ERF016, ERF6 and ORA59 negatively regulate RPW8.1‐mediated cell death and defense responses. Fig. S13 Phenotypic analysis of ERF6, ERF016 and ORA59 knockout mutants in Col‐gl. Fig. S14 The expression pattern of RPW8.1 upon ACC treatment. Methods S1 Yeast two‐hybrid (Y2H) assays. Methods S2 Bimolecular ﬂuorescence complementation (BiFC) assay. Methods S3 Determination of ethylene biosynthesis rates and electrolyte leakage measurements. Methods S4 Leaf senescence assay. Methods S5 RNA extraction and reverse transcription quantitative PCR (RT‐qPCR). Methods S6 Pathogen inoculation and microscopy and analysis. Methods S7 Bacterial growth assays. Methods S8 Luciferase (LUC) reporter assays in Nicotiana benthamiana. Methods S9 Yeast one‐hybrid (Y1H) assays. Methods S10 Protein expression and purification. Methods S11 CRISPR/Cas9 plasmids construction and mutant screening. Table S1 Primers and probes used in this study. Please note: Wiley Blackwell are not responsible for the content or functionality of any Supporting Information supplied by the authors. Any queries (other than missing material) should be directed to the New Phytologist Central Office. [file NPH-229-516-s001.pdf]

New Phytologist **Supporting Information**

Article title: **RPW8.1 enhances the ethylene-signaling pathway to feedback attenuate its mediated cell death and disease resistance in *Arabidopsis***

Authors: Zhi-Xue Zhao, Qin Feng, Peng-Qiang Liu, Xiao-Rong He, Jing-Hao Zhao, Yong-Ju Xu, Ling-Li Zhang, Yan-Yan Huang, Ji-Qun Zhao, Jing Fan, Yan Li, Shunyuan Xiao, Wen-Ming Wang

Article acceptance date: 27 July 2020

The following Supporting Information is available for this article:

Fig. S1 RPW8.1 interacts with itself.
Fig. S2 RPW8.1 specifically interacts with aa 38~175 of ACO4 in yeast.
Fig. S3 The *aco4* mutant compromised in ethylene production.
Fig. S4 Expression patterns of ethylene-related genes upon ACC treatment.
Fig. S5 Expression levels of *RPW8.1* in R1Y4 at two different developmental stages.
Fig. S6 *ACO4* negatively impacts RPW8.1-mediated cell death and defense responses.
Fig. S7 Ethylene signaling plays a negative role in RPW8.1-mediated cell death and defense responses.
Fig. S8 ORA59 binds to the truncated fragments of the *RPW8.1* promoter in yeast.
Fig. S9 ORA59 directly binds to the *RPW8.1* promoter.
Fig. S10 Transcriptional changes of *ERF6*, *ERF016* and *ORA59* in response to powdery mildew infection.
Fig. S11 Mutation sites of *ERF016*, *ERF6* and *ORA59* in R1Y4 and Col-*gl*.
Fig. S12 *ERF016*, *ERF6* and *ORA59* negatively regulate RPW8.1-mediated cell death and defense responses.
Fig. S13 Phenotypic analysis of *ERF6*, *ERF016* and *ORA59* knockout mutants in Col-*gl*.
Fig. S14 The expression pattern of *RPW8.1* upon ACC treatment.

Table S1. The primers and probes used in this study.

Methods S1 Yeast two-hybrid (Y2H) assays

Methods S2 Bimolecular fluorescence complementation (BiFC) assay

Methods S3 Determination of ethylene biosynthesis rates and electrolyte leakage measurements

Methods S4 The leaf senescence assay

Methods S5 RNA extraction and reverse transcription quantitative PCR (RT-qPCR)

Methods S6 Pathogen inoculation and microscopy & analysis

Methods S7 Bacterial growth assays

Methods S8 Luciferase (LUC) reporter assays in *Nicotiana benthamiana*

Methods S9 Yeast one-hybrid (Y1H) assays

Methods S10 Protein expression and purification

Methods S11 CRISPR/Cas9 plasmids construction and mutant screening

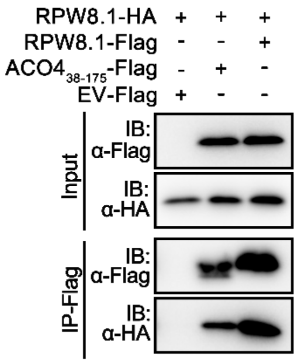

Fig. S1 RPW8.1 interacts with itself.

In vivo Co-IP assay. HA-tagged RPW8.1 was co-expressed with Flag-tagged $ACO4_{38\text{-}175}$ or RPW8.1 in *N. benthamiana* leaves. The pair of Flag empty vector (EV-Flag) and HA-tagged RPW8.1 was used as a negative control. Total proteins were extracted and subjected to immunoprecipitation of $ACO4_{38\text{-}175}$ or RPW8.1 protein by the Flag antibody (α-Flag), followed by immunoblot analysis with the HA antibody (α-HA). The input proteins were analyzed with α-HA and α-Flag. The experiments were repeated for three times with similar results.

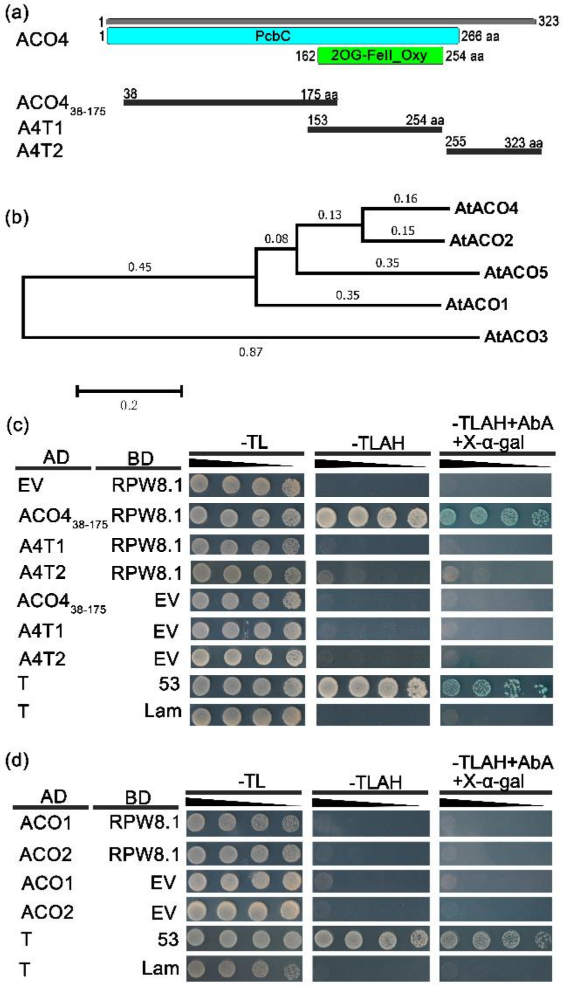

Fig. S2 RPW8.1 specifically interacts with aa 38~175 of ACO4 in yeast.

(a) Schematic illustration of ACO4 and three indicated mutants containing different regions of ACO4 used in yeast two-hybrid (Y2H). The conserved motifs of ACO4 were shown in blue (PcbC, Isopenicillin N synthase) or green (2OG-FeII_Oxy). Numbers indicate the position of amino acids (aa). (b) Phylogram of ACO family members in *Arabidopsis*. The phylogenetic tree was made using the neighbor-joining tree method in MEGA version 7. Amino acid residue sequences of ACOs were downloaded from TAIR (https://www.arabidopsis.org/). AtACO1, AT2G19590; AtACO2, AT1G62380; AtACO3, AT2G05710; AtACO4, AT1G05010; AtACO5, AT1G77330. (c, d) Y2H analysis shows the interaction between RPW8.1 and $ACO4_{38\text{-}175}$ (c), but not between RPW8.1 and ACO1 or ACO2 (d). Full-length *RPW8.1* was translationally fused to the Gal4 DNA-binding domain (BD) of the pGBKT7 destination vector and the fusion protein serves as a bait. *ACO1*, *ACO2* and *ACO4* truncated mutants were fused to the Gal4 activation domain (AD) of the

pGADT7 destination vector and the fusion proteins serves as preys. Yeast cells grown on SD/-Trp/-Leu medium (-TL) indicated correct co-transformation and co-expression of different combinations of empty vectors and recombinant plasmids. Interactions were demonstrated by the growth of yeast cells on selective media SD/-Ade/-His/-Leu/-Trp (-TLAH), and -TLAH media supplemented with X-α-gal (40 μg ml^{-1}) and AbA (200 ng ml^{-1}). The pair of pGBKT7-53 (53) and pGADT7-T (T) was used as a positive control, pGBKT7-Lam (Lam) and pGADT7-T (T) was used as a negative control. Plates were photographed after 3 days.

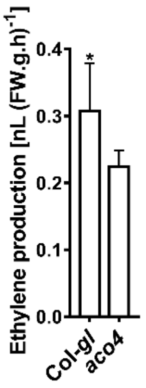

Fig. S3 The *aco4* mutant compromised in ethylene production.

Ethylene biosynthesis rate assay. Seedlings of 2-week-old Col-*gl* and *aco4* mutant were used to measure the ethylene biosynthesis rates. Error bars indicated standard deviation (SD) (n=3). The asterisk (*) above the bar indicates significant differences (P<0.05) determined by Student's *t*-test.

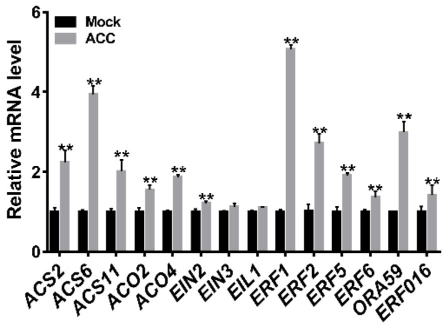

Fig. S4 Expression patterns of ethylene-related genes upon ACC treatment.

Reverse transcription quantitative PCR (RT-qPCR) assay. The relative expression levels of the indicated genes upon ACC (100 μM) treatment were calculated relatively to that of Mock treatment. *ACT2* was used as an internal control. Error bars indicated SD (n=3). Asterisks (**) above the bars indicate significant differences (P<0.01) determined by Student's *t*-test.

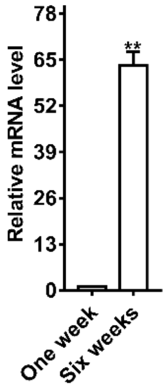

Fig. S5 Expression levels of *RPW8.1* in R1Y4 at two different developmental stages.

Reverse transcription quantitative PCR (RT-qPCR) analysis was used to determine the relative expression levels of *RPW8.1* in R1Y4 plants grown on the 1/2 MS medium at the two different developmental stages. *ACT2* was used as an internal control. Error bars indicate standard deviation (SD) (n=3). The asterisks (**) above the bars indicate significant differences (P<0.01) determined by Student's *t*-test.

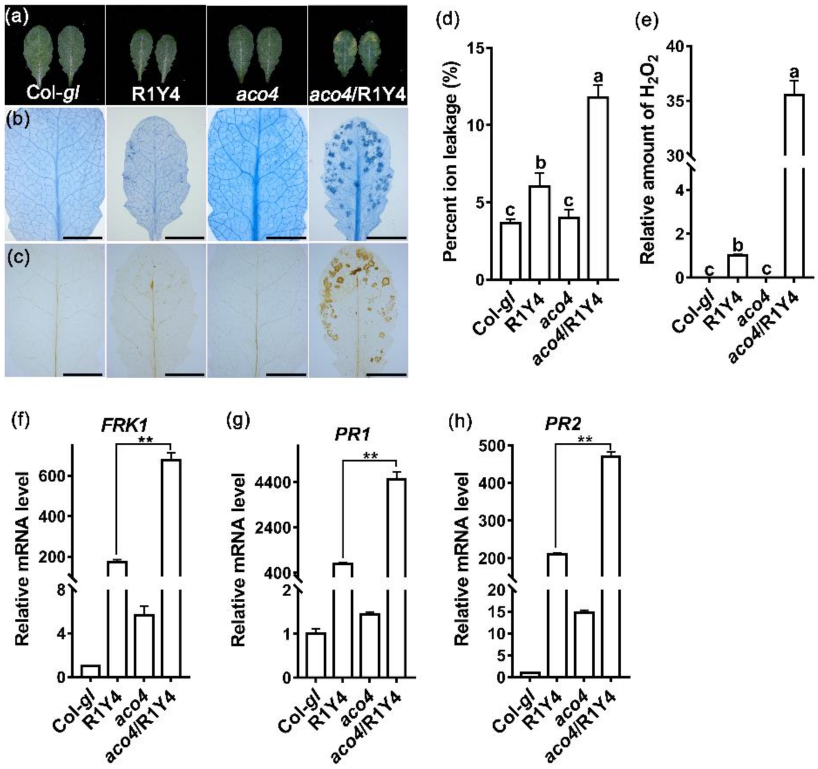

Fig. S6 *ACO4* negatively impacts RPW8.1-mediated cell death and defense responses.

(a) A comparison of representative leaves from the indicated lines. (b, c) Representative leaves stained by trypan blue (b) and 3,3'-diaminobenzidine (DAB) (c), showing cell death and H_2O_2 accumulation in the indicated lines, respectively. Note that there is no any cell death in Col-*gl* and *aco4*, but some clusters of cell death in R1Y4 and massive clusters of cell death in *aco4*/R1Y4. Scale bars, 5 mm. (d) Electrolyte leakage analysis showing the conductivity of the leaves from the indicated lines. Leaves from the indicated lines were subjected to electrolyte leakage analysis at 6-week-old with three biological replicates. Error bars indicate standard deviation (SD) (n=3). Different letters above the bars indicate significant differences (P<0.01) determined by One-way ANOVA. (e) Quantitative analysis of the H_2O_2 accumulation in the leaves shown in (c) using Image J. Four independent leaves from each indicated line were assessed. Error bars indicate SD (n=4). Different letters above the bars indicate significant differences (P<0.01) determined by One-way ANOVA. (f, g, h) Reverse transcription quantitative PCR (RT-qPCR) analysis shows the relative expressions of *FRK1* (f), *PR1* (g) and *PR2* (h) in the indicated lines. Total RNA was extracted form 6-week-old plants. The expression level of each gene was calculated relative to that of Col-*gl*. *ACT2* was used as an internal control. Error bars indicate SD (n=3). Asterisks (**) above

the bars indicate significant differences ($P<0.01$) determined by Student's *t*-test.

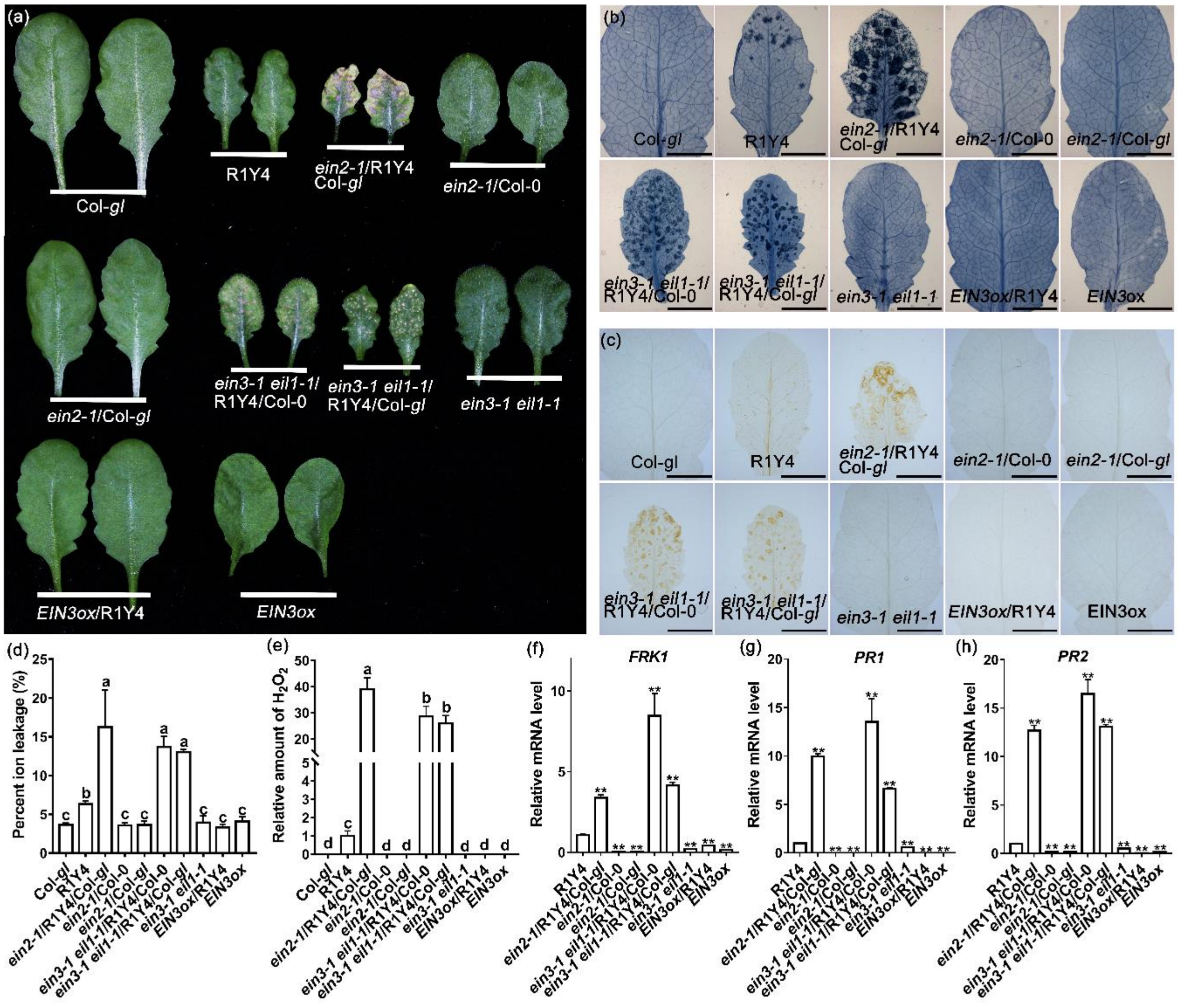

Fig. S7 Ethylene signaling plays a negative role in RPW8.1-mediated cell death and defense responses.

(a) A comparison of representative leaves from the indicated lines. (b, c) Representative leaves stained by trypan blue (b) and 3,3'-diaminobenzidine (DAB) (c), showing cell death and H_2O_2 accumulation in the indicated lines, respectively. Scale bars, 5 mm. (d) Electrolyte leakage analysis showing the conductivity of the leaves from the indicated lines. Leaves from the indicated lines were subjected to electrolyte leakage analysis at 6-week-old with three biological replicates. Error bars indicate standard deviation (SD) (n=3). Different letters above the bars indicate significant differences (P<0.01) determined by One-way ANOVA. (e) Quantitative analysis of H_2O_2 accumulation in the leaves shown in (c) using Image J. Four independent leaves from each indicated line were assessed. Error bars indicated SD (n=4). Different letters above the bars indicate significant differences (P<0.01) determined by One-way ANOVA. (f, g, h) Reverse

transcription quantitative PCR (RT-qPCR) analysis shows the relative expressions of *FRK1* (f), *PR1* (g) and *PR2* (h) in the indicated lines at 6-week-old. The expression level of each gene was calculated relative to that of R1Y4. *ACT2* was used as an internal control. Error bars indicated SD (n=3). Asterisks (**) above the bars indicate significant differences (P<0.01) determined by Student's *t*-test.

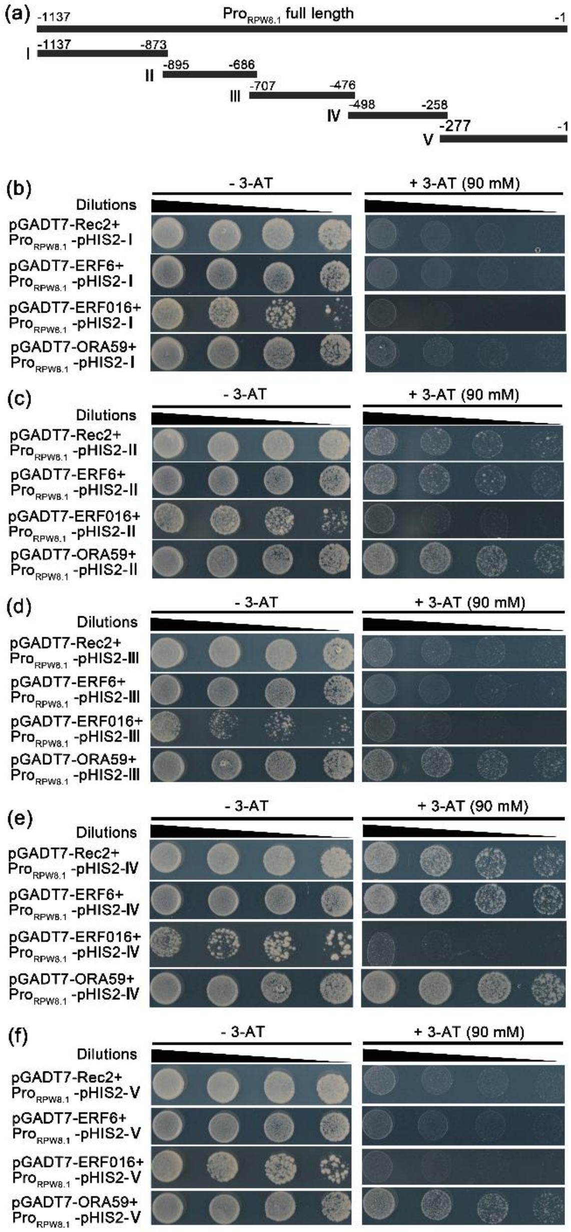

Fig. S8 ORA59 binds to truncated fragments of the *RPW8.1* promoter in yeast.

(a) Schematic representation of the five truncated fragments containing different regions of the *RPW8.1* promoter. Numbers indicate the minus position of base-pairs upstream of A(TG). Five truncated mutants were generated, i.e., I (-1137 to -873 bp), II (-895 to -686 bp), III (-707 to -476 bp), IV (-498 to -258 bp) and V (- 277 to -1 bp). (b, c, d, e, f) Yeast one-hybrid (Y1H) assay tested the interaction between different truncated fragments of the *RPW8.1* promoter and ERF6, ERF016 or ORA59. The truncated fragments of the *RPW8.1* promoter were fused to the pHIS2 vector.

ERF6, ERF016 and ORA59 were fused to the pGADT7 vector. Yeast cells grown on the selective medium SD/-Trp/-Leu/-His supplemented with 90 mM 3-amino-1,2,4-triazole (3-AT) indicated the interactions of the indicated combinations of the constructs. The pairs of pGADT7-Rec2 and pHIS2 vector expressing different truncated mutants were used as negative controls. Plates were photographed after 3 days.

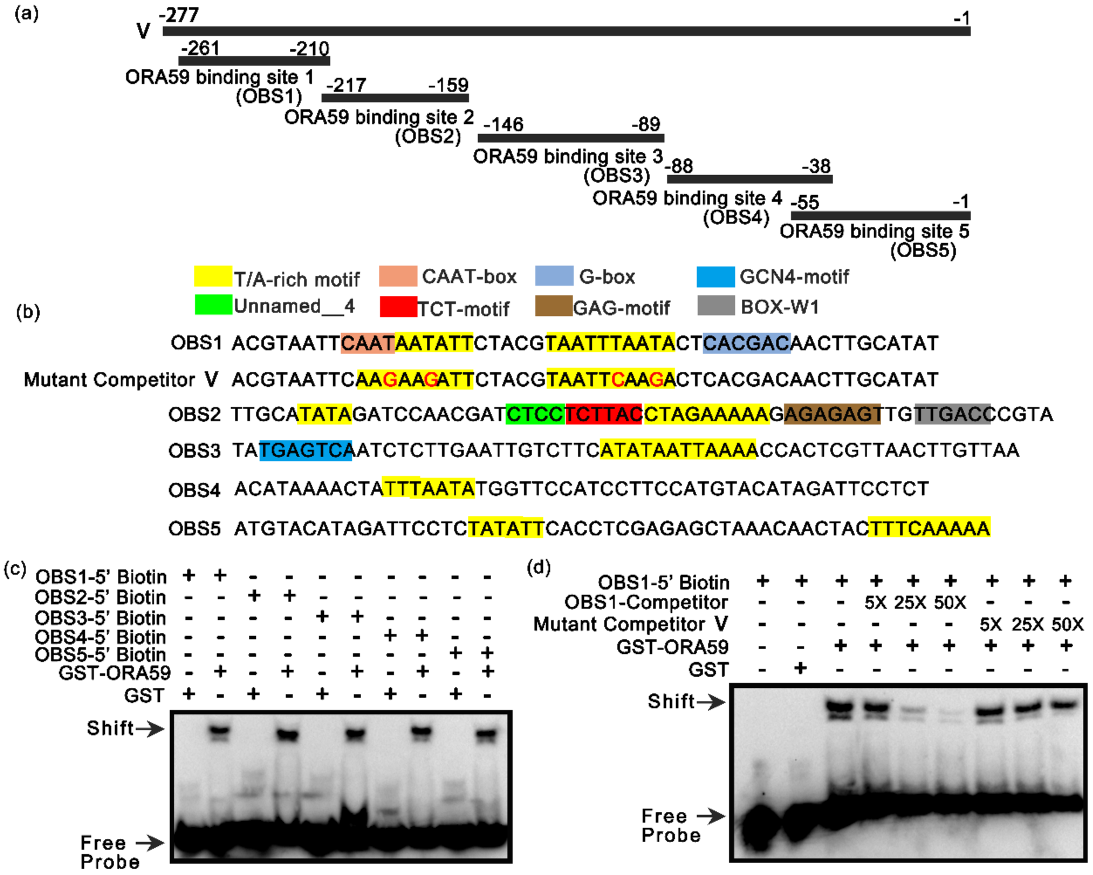

Fig. S9 ORA59 directly binds to the *RPW8.1* promoter.

(a) Schematic representation of the five fragments of *RPW8.1* promoter from -277 bp to -1 bp named ORA59 binding site 1 (OBS1), OBS2, OBS3, OBS4 and OBS5. Numbers indicate the minus position of base-pairs up-stream of A(TG). (b) Sequences of OBS1, OBS2, OBS3, OBS4 and OBS5. Biotinylated probes were synthesized based on the sequences of the truncated fragments. Mutant competitor V acts as a mutant competitor of OBS1. T/A-rich motif is highlighted in yellow; others are highlighted in different colors. Note that a T/A-rich motif is discovered in all five fragments. (c) Electrophoresis mobility shift assay (EMSA) showing that GST-ORA59 fusion protein binds to the T/A-rich motif containing regions of the *RPW8.1* promoter. GST was used as a control. (d) EMSA showing that GST-ORA59 fusion protein possesses T/A-rich motif specific binding activity. OBS1-competitor and the mutant competitor V oligonucleotides were used for competitive binding in 5-, 25-, 50-fold excess of the biotinylated probe. Charge-coupled device (CCD) camera was used for taking photos.

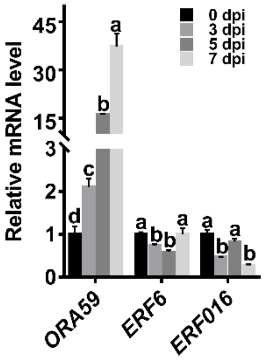

Fig. S10 Transcriptional changes of *ERF6*, *ERF016* and *ORA59* in response to powdery mildew infection.

Reverse transcription quantitative PCR (RT-qPCR) analysis was used to determine the relative expression levels of *ERF6*, *ERF016* and *ORA59* in Col-*gl* upon powdery mildew infection. Samples were collected at the indicated time points. *ACT2* was used as an internal control. Error bars indicated standard deviation (SD) (n=3). Different letters above the bars indicate significant differences (P<0.01) determined by One-way ANOVA.

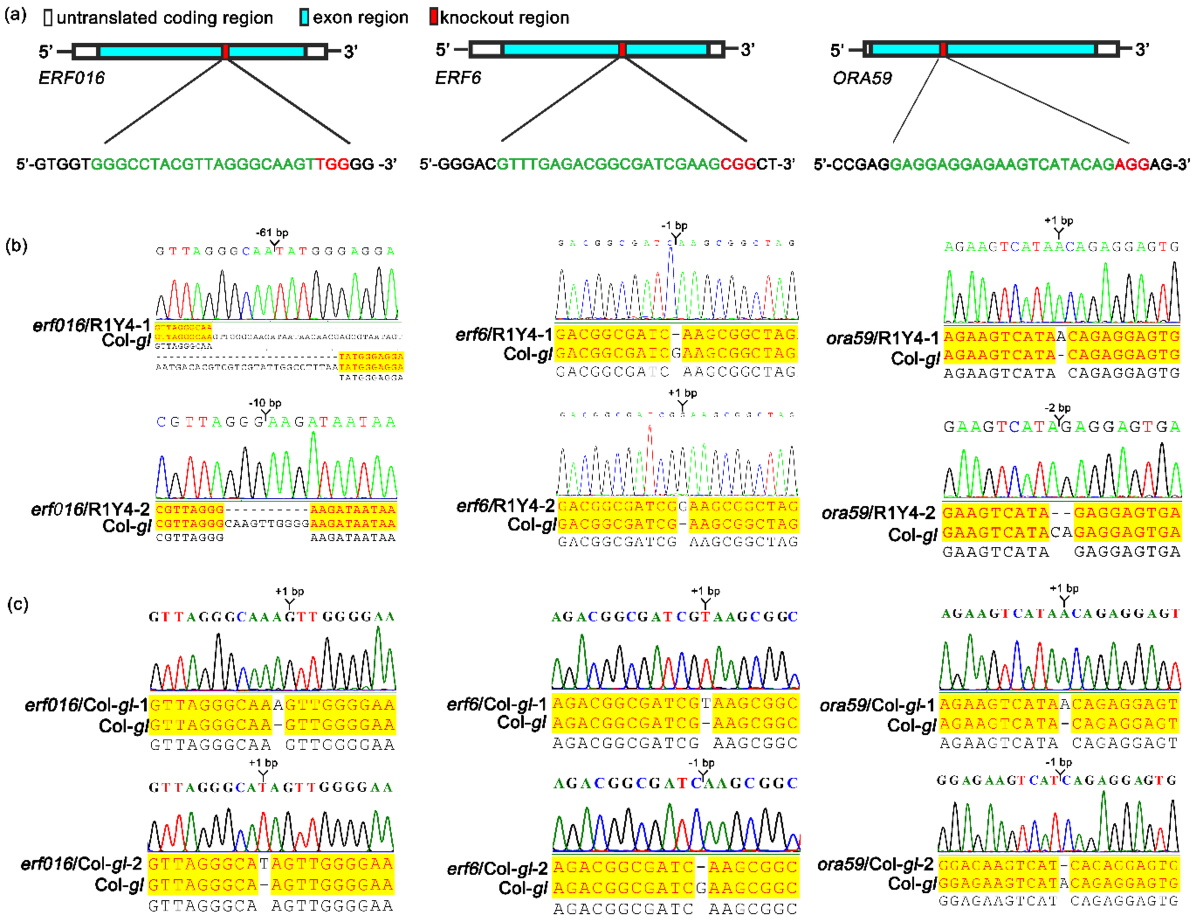

Fig. S11 Mutation sites of *ERF016*, *ERF6* and *ORA59* in R1Y4 and Col-*gl*.

(a) Schematics of the target sites for CRISPR/Cas9 editing in *ERF016*, *ERF6* and *ORA59*. Green, sgRNA targeting sequences; Red, protospacer adjacent motif (PAM). (b, c) The specific DNA mutations of each knockout lines confirmed by PCR-based sequencing. Two independent mutants were generated for each gene in R1Y4 (b) and Col-*gl* (c) backgrounds, i.e., *erf016/R1Y4-1* and *erf016/R1Y4-2, erf6/R1Y4-1* and *erf6/R1Y4-2, ora59/R1Y4-1* and *ora59/R1Y4-2* in the R1Y4 background; *erf016/*Col-*gl-1* and *erf016/*Col-*gl-2, erf6/*Col-*gl-1* and *erf6/*Col-*gl-2, ora59/*Col-*gl-1* and *ora59/*Col-*gl-2* in the Col-*gl* background. Numbers indicate the deleted or inserted bases in the mutants. Among them, *erf016*/R1Y4-1 carried a 61-bp deletion resulting in frame shift at the codon for amino acid residue (aa) 134 that led to an early stop codon after aa 140 (Leucine); *erf016*/R1Y4-2 carried a 10-bp deletion resulting in frame shift at the codon for aa 133 that led to an early stop codon after aa 138 (Arginine); *erf6*/R1Y4-1 carried a 1-bp deletion resulting in frame shift at the codon for amino acid residue (aa) 172 that led to an early stop codon after aa 190

(Phenylalanine); *erf6*/R1Y4-2 carried a 1-bp insertion resulting in frame shift at the codon for aa 172 that led to an early stop codon after aa 174 (Glycine); *ora59*/R1Y4-1 carried a 1-bp insertion resulting in frame shift at the codon for amino acid residue (aa) 82 that led to an early stop codon after aa 81 (Serine); *ora59*/R1Y4-2 carried a 2-bp deletion resulting in frame shift at the codon for aa 82 that led to an early stop codon after aa 81 (Serine); *erf016*/Col-*gl*-1 carried a 1-bp insertion resulting in frame shift at the codon for amino acid residue (aa) 134 that led to an early stop codon after aa 137 (Arginine); *erf016*/Col-*gl*-2 carried a 1-bp insertion resulting in frame shift at the codon for aa 133 that led to an early stop codon after aa 137 (Arginine); *erf6*/Col-*gl*-1 carried a 1-bp insertion resulting in frame shift at the codon for amino acid residue (aa) 172 that led to an early stop codon after aa 174 (Glycine); *erf6*/Col-*gl*-2 carried a 1-bp deletion resulting in frame shift at the codon for aa 172 that led to an early stop codon after aa 190 (Phenylalanine); *ora59*/Col-*gl*-1 carried a 1-bp insertion resulting in frame shift at the codon for amino acid residue (aa) 82 that led to an early stop codon after aa 81 (Serine); *ora59*/Col-*gl*-2 carried a 1-bp deletion resulting in frame shift at the codon for aa 82 that led to an early stop codon after aa 84 (Glutamic acid).

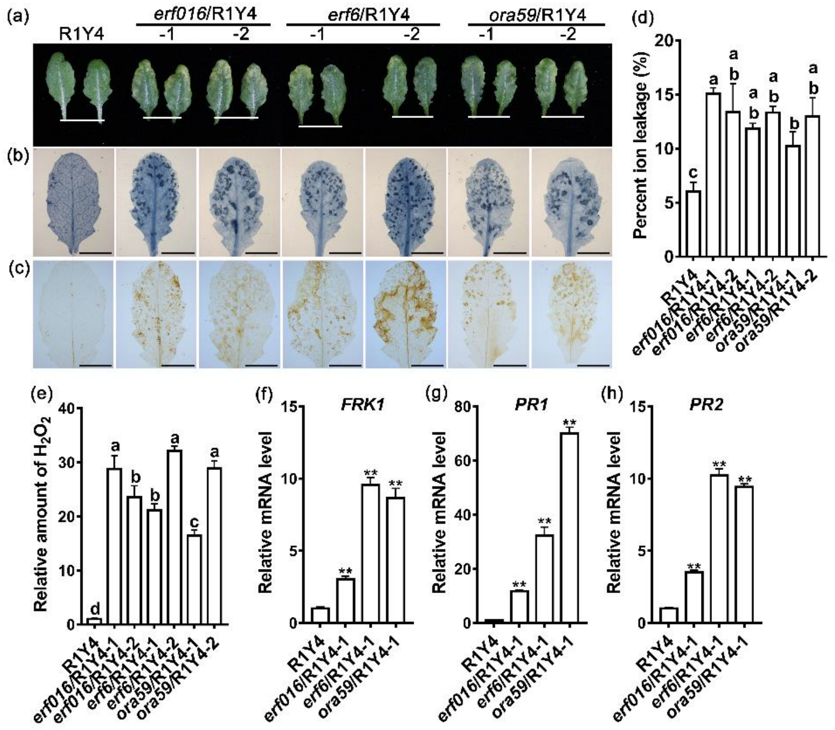

Fig. S12 *ERF016*, *ERF6* and *ORA59* negatively regulate RPW8.1-mediated cell death and defense responses.

(a) A comparison of representative leaves from the indicated lines. (b, c) Representative leaves stained by trypan blue (b) and 3,3'-diaminobenzidine (DAB) (c) showing cell death and H_2O_2 accumulation in the indicated lines, respectively. Scale bars, 5 mm. (d) Electrolyte leakage analysis showing the conductivity of the leaves from the indicated lines. Leaves from the indicated lines were subjected to electrolyte leakage analysis at 6-week-old plants with three biological replicates. Error bars indicate standard deviation (SD) (n=3). Different letters above the bars indicate significant differences (P<0.01) determined by One-way ANOVA. (e) Quantitative analysis of H_2O_2 accumulation in the leaves shown in (c) using Image J. Four independent leaves from each indicated line were assessed. Error bars indicate SD (n=4). Different letters above the bars indicate significant differences (P<0.01) determined by One-way ANOVA. (f, g, h) Reverse transcription quantitative PCR (RT-qPCR) analysis showing the relative expressions of *FRK1* (f), *PR1* (g) and *PR2* (h) in the indicated lines at 6-week-old. The expression level of each gene was calculated relative to that of R1Y4. *ACT2* was used as an internal control. Error bars indicate SD (n=3). Asterisks (**) above the bars indicate significant differences (P<0.01) determined by Student's *t*-test.

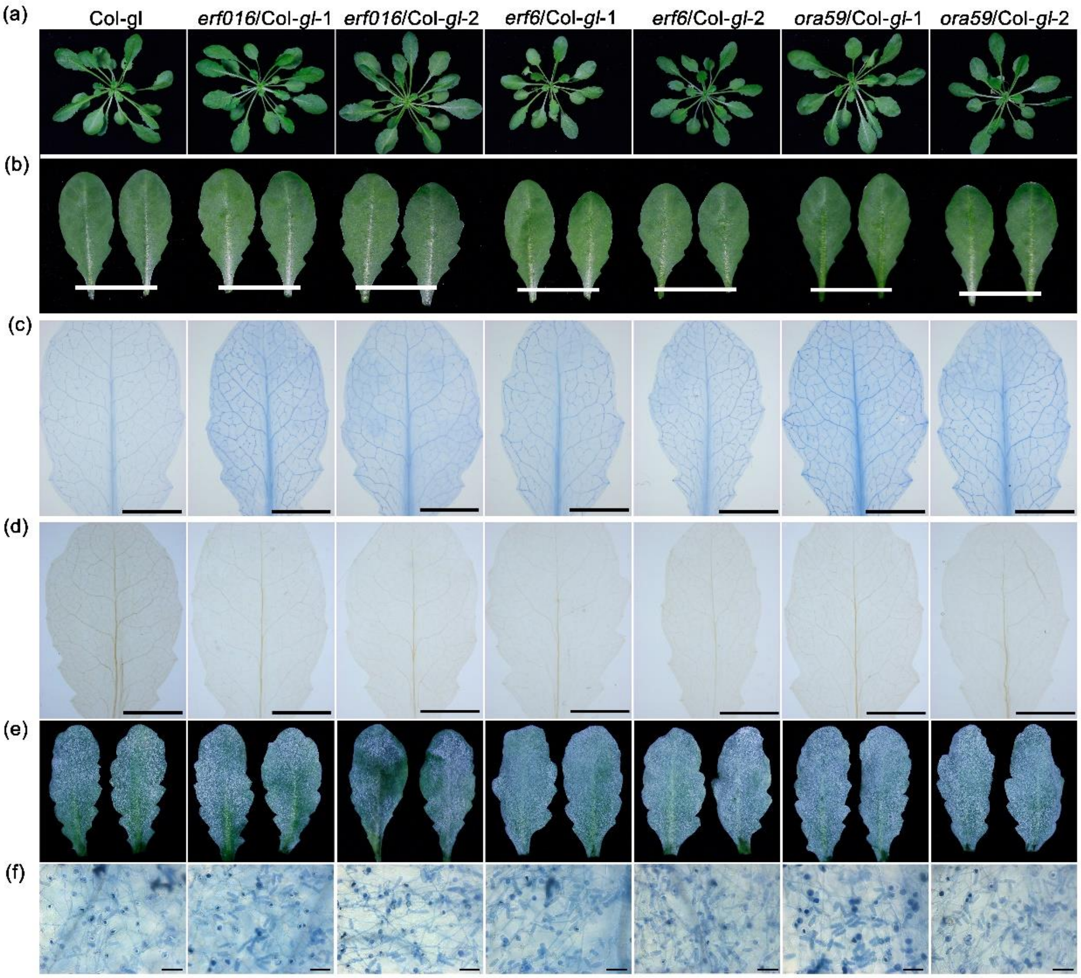

Fig. S13 Phenotypic analysis of *ERF6*, *ERF016* and *ORA59* knockout mutants in Col-*gl*.

(a) Plant phenotypes of 6-week-old *efr016*, *erf6* and *ora59* mutants in the Col-*gl* background. (b) A comparison of representative leaves from the indicated lines. (c, d) Representative leaves stained by trypan blue (c) and 3,3'-diaminobenzidine (DAB) (d) from the indicated lines. Note that there are no any cell death and H_2O_2 accumulation in the indicated lines. Scale bars, 5 mm. (e) Representative leaves showing the disease phenotypes of powdery mildew at 10 days post inoculation (dpi) in the indicated lines. (f) Representative infected leaf sections showing the fungal infection and sporulation of powdery mildew stained with trypan blue at 10 dpi from the indicated lines. Scale bars, 100 μm.

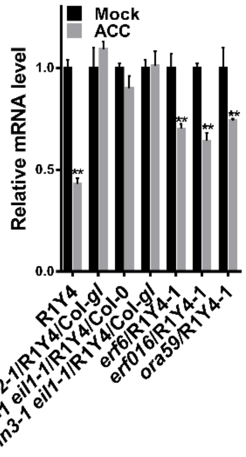

Fig. S14 The expression pattern of *RPW8.1* upon ACC treatment.

Reverse transcription quantitative PCR (RT-qPCR) assay was used to determine the expressions levels of *RPW8.1* in the indicated plants upon ACC (100 μM) treatment, which were calculated relative to that of Mock treatment. *ACT2* was used as an internal control. Error bars indicate SD (n=3). Asterisks (**) above the bars indicate significant differences (P<0.01) determined by Student's *t*-test.

Table S1. The primers and probes used in this study.

Primers	Sequence (5'-3')	Objective	Note
NbEF-1a-F	CTGATTATTGACTCCACCACTG	RT-qPCR	
NbEF-1a-R	CATCTTGTTACAGCAGCAAATC	RT-qPCR	
RT-NbACS1-F	GCAGCAGGAGCAAACTTCAG	RT-qPCR	
RT-NbACS1-R	AGTCCTGACCAAAGGTGAACTC	RT-qPCR	
RT-NbACS2-F	GTTATGGCTGGTGGTGCAAC	RT-qPCR	
RT-NbACS2-R	TCTCTGTCCAAAGTGGTGCC	RT-qPCR	
RT-NbACS4-F	AAACCAGGCGTCCAGATCAG	RT-qPCR	
RT-NbACS4-R	CTGGACGCTTGTTTGTCGAC	RT-qPCR	
NbACO-RT-F	GGATTTGCTCTGTGAAAATCTT	RT-qPCR	
NbACO-RT-R	AGGATTATGCCACCAGCGTC	RT-qPCR	
NbEIN2-F-RT	GGCAGCAAAAATCAAGCCCCC	RT-qPCR	
NbEIN2-R-RT	CTGAAAGGGGCTGCTGAAG	RT-qPCR	
RT-NbERF1-F	AAAGTGCGAGTCCGAAGAGG	RT-qPCR	
RT-NbERF1-R	TCTCCGCTGAAAATGTCCCC	RT-qPCR	
RT-NbERF3-F	GCAGAACGCGCCCTTCGAG	RT-qPCR	
RT-NbERF3-R	ATCGGCGTTACATGGTAAGGCT	RT-qPCR	
ACT2F	GTGATGAAGCACAATCCAAGAGA	RT-qPCR	
ACT2R	TCAGTAAGGTCACGTCCAGCAA	RT-qPCR	
RPW8.1 P-F for RT	ATAGGGGCTGTTCTTGGAGTTG	RT-qPCR	
RPW8.1 P-R for RT	TTGTCTGCGTCTGAGTTTCGG	RT-qPCR	
ACS2-F for RT	TCATGGGAAAAGCTAGAGGTGGAAG	RT-qPCR	
ACS2-R for RT	TCAACGGTTAATTTGAAATTGTCGG	RT-qPCR	
AtACS6-RT-F	AAACCGATGGCTGCAACAACTATGA	RT-qPCR	
AtACS6-RT-R	TAAGTCTGTGCACGGACTAGCGGAG	RT-qPCR	
AtACS11-RT-F	CTGGTTTCGGGTCTAAAGGAAGCGG	RT-qPCR	
AtACS11-RT-R	AATGACACGATGAGCCTGGAGAGATGTT	RT-qPCR	
AtACO2-RT-F	GGATGTCGGTTGCATCGTTTTA	RT-qPCR	
AtACO2-RT-R	TACGGCTGCTGTAGGATTCAGTTC	RT-qPCR	
AtACO4-RT-F	CTACCTCAAGCACCTTCCCG	RT-qPCR	
AtACO4-RT-R	TTCTCGCACAGCAGATCCAG	RT-qPCR	
EIN2-RT-F	TGGAGGAGGAGGGTATGGTG	RT-qPCR	
EIN2-RT-R	CACCAAACTGCTCAAAGGGC	RT-qPCR	
EIN3-RT-F	CCGATTGGACCGACTCCTCATAC	RT-qPCR	
EIN3-RT-R	ATAGCAAGCCAGGTAGCACTCTC	RT-qPCR	
EIl1-RT-F	CAACACCGCATACGCTTCAG	RT-qPCR	
EIl1-RT-R	GCCACCACTCTTCATTCCCA	RT-qPCR	
ERF1-RT-1	TTAATTCAGTCCCCATTCTC	RT-qPCR	
ERF1-RT-2	CCAAGTCCCACTATTTTCAG	RT-qPCR	

ERF2-RT-1	TGATCCGGCGAAGAATGGAG	RT-qPCR	
ERF2-RT-2	CAATAAAGCGCGGGAACCAC	RT-qPCR	
ERF5-RT-1	TACTCGACGAGGCTTCTCCT	RT-qPCR	
ERF5-RT-2	GGGCGAAAGAAGAGGACGAT	RT-qPCR	
ERF016-RT-F	GGGAAAATGGGTGGCTGAGA	RT-qPCR	
ERF016-RT-R	GGGGCCACGAAGACAGTAAA	RT-qPCR	
ORA59-RT-F	CCGAGGAGGAGGAGAAGTCAT	RT-qPCR	
ORA59-RT-R	GTGTCGAATGTCCCAAGCCA	RT-qPCR	
ERF6-RT-F	ACTACTGCCACCACCAATCG	RT-qPCR	
ERF6-RT-R	TGAACAGTAACGCGAGGAGG	RT-qPCR	
PR1-RT-F	TACGCAGAACAACTAAGAGG	RT-qPCR	
PR1-RT-R	TCGTTCACATAATTCCCACG	RT-qPCR	
PR2-RT-F	TCGATGAGAATAAGAAGGAACCAAC	RT-qPCR	
PR2-RT-R	ATAACAACATACTACACGCTGAAAG	RT-qPCR	
FRK1F	TCTGAAGAATCAGCTCAAGGC	RT-qPCR	
FRK1R	TGTTGGCTTCACATCTCTGTG	RT-qPCR	
RPW8.1-pGBKT7-EcoR1-F	TGGCCATGGAGGCCGAATTCATGCCGATTGGTGAGCTTGCGATA	Make construct	For Y2H analysis
RPW8.1-pGBKT7-BamH1-R	CGCTGCAGGTCGACGGATCCTCAAGCTCTTATTTTACTACAAGC	Make construct	For Y2H analysis
RPW8.2-pGBKT7-EcoR1-F	TGGCCATGGAGGCCGAATTCATGATTGCTGAGGTTGCCGCAG	Make construct	For Y2H analysis
RPW8.2-pGBKT7-BamH1-R	CGCTGCAGGTCGACGGATCCTCAAGAATCATCACTGCAGAACG	Make construct	For Y2H analysis
ACO4F-pGADT7-EcoR1-F	CCATGGAGGCCAGTGAATTCATGGAGAGTTTCCCGATCATCAA	Make construct	For Y2H analysis
ACO4F-pGADT7-BamH1-R	AGCTCGAGCTCGATGGATCCTCACGCAGTGGCCAATGGTCCA	Make construct	For Y2H analysis
ACO4$_{38-175}$-pGADT7-EcoR1-F	CCATGGAGGCCAGTGAATTCATGAACCATGGGATTTCACTCGA	Make construct	For Y2H analysis
ACO4$_{38-175}$-pGADT7-BamH1-R	AGCTCGAGCTCGATGGATCCTCATCGGAGACCCTTGACTAGGT	Make construct	For Y2H analysis
A4T1-pGADT7-EcoR1-F	CCATGGAGGCCAGTGAATTCATGACTTTTGGAACCAAAGTCAG	Make construct	For Y2H analysis
A4T1-pGADT7-BamH1-R	AGCTCGAGCTCGATGGATCCTCACGGATTATAGAATGATGCGA	Make construct	For Y2H analysis
A4T2-pGADT7-EcoR1-F	CCATGGAGGCCAGTGAATTCATGGGAAGCGACTCTGTTATTTTTCC	Make construct	For Y2H analysis
A4T2-pGADT7-BamH1-R	AGCTCGAGCTCGATGGATCCTCACGCAGTGGCCAATGGTC	Make construct	For Y2H analysis
ACO2-pGADT7-EcoR1-F	CCATGGAGGCCAGTGAATTCATGGAGAAGAACATGAAGTTTC	Make construct	For Y2H analysis
ACO2-pGADT7-BamH1-R	AGCTCGAGCTCGATGGATCCTTAGAAAGTCTCTACGGCTGCT	Make construct	For Y2H analysis
ACO1-pGADT7-EcoR1-F	CCATGGAGGCCAGTGAATTCATGGTTTTGATCAAAGAGAGAGAG	Make construct	For Y2H analysis

ACO1-pGADT7-BamH1-R	AGCTCGAGCTCGATGGATCCCTAGGCTGAATCCGCATTTCCCA	Make construct	For Y2H analysis
RPW8.1-BamH1-F	CGGGGATCCATGCCGATTGGTGAGCTTGCGAT	Make construct	For BiFC analysis
RPW8.1-Sal1-106-R	GCCGTCGACTCAAGCTCTTATTTTACTACAAGCA	Make construct	For BiFC analysis
RPW8.1-Sal1-104-R	GCCGTCGACAGCTCTTATTTTACTACAAGCA	Make construct	For BiFC analysis
$ACO4_{38\text{-}175}$-BamH1-F	CGGGGATCCATGAACCATGGGATTTCACTCGAGC	Make construct	For BiFC analysis
$ACO4_{38\text{-}175}$-Sal1-106-R	GCCGTCGACTTATCGGAGACCCTTGACTAGG	Make construct	For BiFC analysis
$ACO4_{38\text{-}175}$-Sal1-104-R	GCCGTCGACTCGGAGACCCTTGACTAGG	Make construct	For BiFC analysis
ACO4-BamH1-F	CGGGGATCCATGGAGAGTTTCCCGATCATCAAT	Make construct	For BiFC analysis
ACO4- Sal1-106-R	GCCGTCGACTCACGCAGTGGCCAATGGTCCA	Make construct	For BiFC analysis
ACO4- Sal1-104-R	GCCGTCGACCGCAGTGGCCAATGGTCCA	Make construct	For BiFC analysis
RPW8.1-BamH1-F	ACAGGATCCATGCCGATTGGTGAGCTTGCGATA	Make construct	For western blot
RPW8.1-Stu1-F	ACAAGGCCTAGCTCTTATTTTACTACAAGC	Make construct	For western blot
ACO4-BamH1-F	ACAGGATCCATGGAGAGTTTCCCGATCATCAA	Make construct	For western blot
ACO4-Stu1-R	ACAAGGCCTCGCAGTGGCCAATGGTCCA	Make construct	For western blot
$ACO4_{38\text{-}175}$-BamH1-F	ACAGGATCCATGAACCATGGGATTTCACTCGA	Make construct	For western blot
$ACO4_{38\text{-}175}$-Stu1-R	ACAAGGCCTTCGGAGACCCTTGACTAGGT	Make construct	For western blot
RPW8.1 Promoter-EcoR1-F	CACGAATTCATTGGTCTCTCAATTTGTTTATTTAGAA	Make construct	For LUC reporter analysis
RPW8.1 Promoter-Kpn1-R	CACGGTACCTTTTTGAAAGTAGTTGTTTAGCTCTC	Make construct	For LUC reporter analysis
EIL1-Kpn1-F	CACGGTACCATGATGATGTTTAACGAGATGGGA	Make construct	For LUC reporter analysis
EIL1-Pst1-R	ACACTGCAGTCAGAACCATATTGATACATCTTGC	Make construct	For LUC reporter analysis
ERF1-Kpn1-F	ACAGGTACCATGGATCCATTTTTAATTCAGTCCC	Make construct	For LUC reporter analysis
ERF1-Spe1-R	CACACTAGTTCACCAAGTCCCACTATTTTCAG	Make construct	For LUC reporter analysis
ORA59-Kpn1-F	ACAGGTACCATGGAATATCAAACTAACTTCTTAAGTG	Make construct	For LUC reporter analysis
ORA59-Spe1-R	CACACTAGTTCAAGAACATGATCTCATAAGCTCT	Make construct	For LUC reporter analysis
ERF2-Kpn1-F	ACAGGTACCATGTACGGACAGTGCAATATAGAATC	Make construct	For LUC reporter analysis

ERF2-Spe1-R	CACACTAGTTTATGAAACCAATAACTCATCAACACGT	Make construct	For LUC reporter analysis
ERF5-Kpn1-F	ACAGGTACCATGGCGACTCCTAACGAAGTATC	Make construct	For LUC reporter analysis
ERF5-Spe1-R	CACACTAGTTCAAACAACGGTCAACTGGGAAT	Make construct	For LUC reporter analysis
ERF6-Kpn1-F	ACAGGTACCATGGCTACACCAAACGAAGTATCA	Make construct	For LUC reporter analysis
ERF6-Spe1-R	CACACTAGTTCAAACAACGGTCAATTGTGGATAA	Make construct	For LUC reporter analysis
ERF016-Kpn1-F	ACAGGTACCATGGATGCATCCCCAAAGTACA	Make construct	For LUC reporter analysis
ERF016-Spe1-R	CACACTAGTTCAGAAATTCCAAAGGTTAATATTGTA	Make construct	For LUC reporter analysis
RPW8.1p-his2-EcoR1-F	ACGACTCACTATAGGGCGAATTCATTGGTCTCTCAATTTGTTTATTTAGAA	Make construct	For Y1H analysis
RPW8.1p-his2-Sac1-R	TCGATTCGCGAACGCGTGAGCTCTTTTTGAAAGTAGTTGTTTAGCTCTC	Make construct	For Y1H analysis
ERF6-rec2-Nde1-F	TACCAGATTACGCTCATATGATGGCTACACCAAACGAAGTATC	Make construct	For Y1H analysis
ERF6-rec2-EcoR1-R	CCACTGCTTGGGTGGAATTCTCAAACAACGGTCAATTGTGGATA	Make construct	For Y1H analysis
ERF016-rec2-Nde1-F	TACCAGATTACGCTCATATGATGGATGCATCCCCAAAGTAC	Make construct	For Y1H analysis
ERF016-rec2-EcoR1-R	CCACTGCTTGGGTGGAATTCTCAGAAATTCCAAAGGTTAATATTG	Make construct	For Y1H analysis
ORA59-rec2-Nde1-F	TACCAGATTACGCTCATATGATGGAATATCAAACTAACTTCTTAA	Make construct	For Y1H analysis
ORA59-rec2-EcoR1-R	CCACTGCTTGGGTGGAATTCTCAAGAACATGATCTCATAAGCT	Make construct	For Y1H analysis
RPW8.1p-his2-Sac1-R1	ATTCGCGAACGCGTGAGCTCGATCAAATCAAATGAGAAGAATCC	Make construct	For Y1H analysis
RPW8.1p-his2-EcoR1-F1	ACTCACTATAGGGCGAATTCGGATTCTTCTCATTTGATTTGATC	Make construct	For Y1H analysis
RPW8.1p-his2-Sac1-R2	ATTCGCGAACGCGTGAGCTCGAAATCTGAATTAGCCCAGAAT	Make construct	For Y1H analysis
RPW8.1p-his2-EcoR1-F2	ACTCACTATAGGGCGAATTCATTCTGGGCTAATTCAGATTTC	Make construct	For Y1H analysis
RPW8.1p-his2-Sac1-R3	ATTCGCGAACGCGTGAGCTCAATACTTTGCATGTTGCAGTATA	Make construct	For Y1H analysis
RPW8.1p-his2-EcoR1-F3	ACTCACTATAGGGCGAATTCTATACTGCAACATGCAAAGTATT	Make construct	For Y1H analysis
RPW8.1p-his2-Sac1-R4	ATTCGCGAACGCGTGAGCTCACGTCGAAGAAGCGAGAGTC	Make construct	For Y1H analysis
RPW8.1p-his2-EcoR1-F4	ACTCACTATAGGGCGAATTCGACTCTCGCTTCTTCGACGT	Make construct	For Y1H analysis
ORA59-pGEX-BamH1-F	TCCAGGGGCCCCTGGGATCCATGGAATATCAAACTAACTTCTTAA	Make construct	For protein expression
ORA59-pGEX-EcoR1-R	TCGAGTCGACCCGGGAATTCTCAAGAACATGATCTCATAAGCT	Make construct	For protein expression

ERF016-DT-BsF	ATATATGGTCTCGATTGGGCCTACGTTAGGGCAAGTGTTTTAGAGCTAGAAATAGC	Make knockout construct	
ERF016-DT-BsR	ATTATTGGTCTCGAAACGGAATCTCCGGAGGATTGTCAATCTCTTAGTCGACTCTAC	Make knockout construct	
ERF6-DT-BsF	ATATATGGTCTCGATTGTTTGAGACGGCGATCGAAGGTTTTAGAGCTAGAAATAGC	Make knockout construct	
ERF6-DT-BsR	ATTATTGGTCTCGAAACTCAAATCCCAGTCATCTATCAATCTCTTAGTCGACTCTAC	Make knockout construct	
ORA59-DT-BsF	ATATATGGTCTCGATTGGCTCTCGCTTATGATCAGGGTTTTAGAGCTAGAAATAGC	Make knockout construct	
ORA59-DT-BsR	ATTATTGGTCTCGAAACCTGTATGACTTCTCCTCCTCAATCTCTTAGTCGACTCTAC	Make knockout construct	
SALK_064286（ACO4）-LP	ATTGTTGGCCACAGTTGTCTC	Mutant identification	
SALK_064286（ACO4）-RP	GAGATGGAGAGTTTCCCGATC	Mutant identification	
OBS1-F	ACGTAATTCAATAATATTCTACGTAATTTAATACTCACGACAACTTGCATAT	Probe	3`Biotin
OBS1-R	ATATGCAAGTTGTCGTGAGTATTAAATTACGTAGAATATTATTGAATTACGT	Probe	3`Biotin
OBS2-F	TTGCATATAGATCCAACGATCTCCTCTTACCTAGAAAAAGAGAGAGTTGTTGACCCGTA	Probe	3`Biotin
OBS2-R	TACGGGTCAACAACTCTCTCTTTTTCTAGGTAAGAGGAGATCGTTGGATCTATATGCAA	Probe	3`Biotin
OBS3-F	TATGAGTCAATCTCTTGAATTGTCTTCATATAATTAAAACCACTCGTTAACTTGTTAA	Probe	3`Biotin
OBS3-R	TTAACAAGTTAACGAGTGGTTTTAATTATATGAAGACAATTCAAGAGATTGACTCATA	Probe	3`Biotin
OBS4-F	ACATAAAACTATTTAATATGGTTCCATCCTTCCATGTACATAGATTCCTCT	Probe	3`Biotin
OBS4-R	AGAGGAATCTATGTACATGGAAGGATGGAACCATATTAAATAGTTTTATGT	Probe	3`Biotin
OBS5-F	ATGTACATAGATTCCTCTATATTCACCTCGAGAGCTAAACAACTACTTTCAAAAA	Probe	3`Biotin
OBS5-R	TTTTTGAAAGTAGTTGTTTAGCTCTCGAGGTGAATATAGAGGAATCTATGTACAT	Probe	3`Biotin
OBS1-Competitor-F	ACGTAATTCAATAATATTCTACGTAATTTAATACTCACGACAACTTGCATAT	Probe	
OBS1-Competitor--R	ATATGCAAGTTGTCGTGAGTATTAAATTACGTAGAATATTATTGAATTACGT	Probe	
OBS2-Competitor-F	TTGCATATAGATCCAACGATCTCCTCTTACCTAGAAAAAGAGAGAGTTGTTGACCCGTA	Probe	
OBS2-Competitor-R	TACGGGTCAACAACTCTCTCTTTTTCTAGGTAAGAGGAGATCGTTGGATCTATATGCAA	Probe	
Mutant Competitor-V-F	ACGTAATTCAAGAAGATTCTACGTAATTCAAGACTCACGACAACTTGCATAT	Probe	
Mutant Competitor-V-R	ATATGCAAGTTGTCGTGAGTCTTGAATTACGTAGAATCTTCTTGAATTACGT	Probe	

Mutant Competitor-I-F	TTGCAGACAGATCCAACGATCTCCTCTTACCTCGAAAAAGAGAGAGTTGTTGACCCGTA	Probe	
Mutant Competitor-I-R	TACGGGTCAACAACTCTCTCTTTTTCGAGGTAAGAGGAGATCGTTGGATCTGTCTGCAA	Probe	
Mutant Competitor-II-F	TTGCATATAGATCCAACGATCTCCTCTTACCTAGAAAAAGACACAGTTGTTGACCCGTA	Probe	
Mutant Competitor-II-R	TACGGGTCAACAACTGTGTCTTTTTCTAGGTAAGAGGAGATCGTTGGATCTATATGCAA	Probe	
Mutant Competitor-III-F	TTGCATATAGATCCAACGATCTCCTCTTACCTAGAAAAAGAGAGAGTTGTACACCCGTA	Probe	
Mutant Competitor-III-R	TACGGGTGTACAACTCTCTCTTTTTCTAGGTAAGAGGAGATCGTTGGATCTATATGCAA	Probe	
Mutant Competitor-IV-F	TTGCATATAGATCCAACGATCTCGACTTACCTAGAAAAAGAGAGAGTTGTTGACCCGTA	Probe	
Mutant Competitor-IV-R	TACGGGTCAACAACTCTCTCTTTTTCTAGGTAAGTCGAGATCGTTGGATCTATATGCAA	Probe	

Methods S1 Yeast two-hybrid (Y2H) assays

Y2H was performed to screen for RPW8.1-interacting proteins. The coding sequences of *RPW8.1* were amplified from the cDNA synthesized from *RPW8.1*'s mRNA in R1Y4 with the gene-specific primer pairs listed in Table S1 and were cloned into the vector pGBKT7 (BD) (Clontech), forming the bait construct. An *Arabidopsis* cDNA library was constructed by cloning the cDNA synthesized from the total RNA extracted from *Golovinomyces cichoracearum* UCSC1 infected *Arabidopsis* leaves into the Y2H vector pGADT7 (AD) (Clontech). This library was screened using RPW8.1-BD as a bait in the *Saccharomyces cerevisiae* strain Y2H Gold (Clontech). Positive clones were selected on the nutrient-deficient (SD/-Leu/-Trp/-His) media, and were further confirmed on the selective media SD/-Ade/-His/-Leu/-Trp. Twenty positive clones were obtained and the cDNA fragments encoding putative RPW8.1-interacting proteins were sequenced to reveal gene identities.

To generate the plasmids for Y2H analysis, we amplified the coding sequences of *RPW8.1* and *RPW8.2* from the cDNA of R1Y4 and R2Y4 (Huang *et al.*, 2019) with the primer pairs indicated in Table S1. The cDNA fragments were cloned into the yeast bait vector pGBKT7 (BD) (Clontech) at the *Eco*RI/*Bam*HI sites. The full length and truncated fragment of *ACO4* were amplified from the cDNA of R1Y4 with the primer pairs indicated in Table S1 and were cloned into the yeast prey vector pGADT7 (AD) (Clontech) at the *Eco*RI/*Bam*HI sites. The pairs of Y2H plasmids were co-transformed into the *S. cerevisiae* strain Y2H Gold (Clontech) according to the user's manual. The pair of pGADT7-T and pGBKT7-53 were used as a positive control, while pGADT7-T and pGBKT7-Lam were used as negative control. Transformants were selected using the nutrient-deficient (SD/-Leu/-Trp) medium at 30 ℃ for 3 days. Then positive transformants were subjected to a 10-fold serial dilution, and 6 μL droplets were placed on selective media SD/-Ade/-His/-Leu/-Trp (-TLAH), and -TLAH media supplemented with X-α-gal (40 μg ml^{-1}) and AbA (200 ng ml^{-1}) to assess protein and protein interaction. The results were obtained after 3 days at 30 ℃, the blue colonies indicated interactions.

Methods S2 Bimolecular fluorescence complementation (BiFC) assay

The coding sequences of *RPW8.1*, *ACO4* and $ACO4_{38\text{-}175}$ without stop codon were amplified from their respective cDNA derived from the mRNA of R1Y4 with the primer pairs indicated in Table S1. They were cloned into the pXY104 (carboxyl (C)-half of YFP, contained an HA tag) vector at

the *Bam*HI/*Sal*I sites to make constructs expressing RPW8.1-YC, ACO4-YC and $ACO4_{38-175}$-YC, respectively. Similarly, the coding sequences of *RPW8.1*, *ACO4* and *$ACO4_{38-175}$* with stop codon were amplified and cloned into pXY106 (amino (N)- half of YFP, contained a Flag tag)) vector at the *Bam*HI/*Sal*I sites to make constructs expressing YN-RPW8.1, YN-ACO4 and YN-$ACO4_{38-175}$, respectively. The recombinant constructs for BiFC assays were transfected into the *Agrobacteria* strain GV3101. The combinations of RPW8.1-YC and YN-ACO4, RPW8.1-YC and YN-$ACO4_{38-175}$, YN- RPW8.1 and ACO4-YC, YN-RPW8.1 and $ACO4_{38-175}$-YC were transiently co-expressed in *Nicotiana benthamiana* leaves by agroinfiltration. The pairs of pXY106 and RPW8.1-cYFP, pXY106 and ACO4-cYFP, pXY106 and $ACO4_{38-175}$-cYFP were used as negative controls. Confocal microscopy (Nikon A1) was used to capture YFP fluorescence and chloroplast auto-fluorescence two days after infiltration.

Methods S3 Determination of ethylene biosynthesis rates and electrolyte leakage measurements

Ethylene production of Col-*gl* and R1Y4 was determined by gas chromatography (GC2014, Shimadzu, Japan) equipped with a flame ionization detector as described previously (Yang *et al.*, 2017; Zhang *et al.*, 2017) after slightly modified. Briefly, 2-week-old seedings without roots were used and placed in a 10 mL penicillin bottle for twenty-four hours with a minimum supply of distilled water, then ethylene in 1 mL gas sample was detected and quantified by the gas chromatography equipped. Ethylene biosynthesis rate was calculated in nL per gram fresh weight per hour [nL $(FW.g.h)^{-1}$].

For electrolyte leakage analysis, the detached leaves were washed in deionized water for 2 hours, the conductivity was measured by pH/Water Quality Analyzer DS-70 (HORIBA). Then both of the leaves and water were autoclaved, the conductivity was measured again after cooling. The ratios of ion leakage before and after autoclave were calculated, the results are used to evaluate the degree of electrolyte leakage.

Methods S4 The leaf senescence assay

The methods used for leaf senescence assay were conducted following modification from the previous report (Li *et al.*, 2013). Briefly, the fifth and sixth rosette leaves from 5-week-old *Arabidopsis* plants were excised and floated on the distilled water with or without 100 µM ACC in the plastic petri dishes and kept at 22 °C under the dark condition for 4 days.

Methods S5 RNA extraction and reverse transcription quantitative PCR (RT-qPCR)

RNA extraction and RT-qPCR assay were performed following a previous report (Zhao *et al.*, 2019). Briefly, total RNA was extracted from the leaves using TRIzol reagent (Invitrogen) and the first strain cDNA was synthesized using ReverTra Ace® qPCR RT Master Mix with gDNA Remover kit (TOYOBO) according to the manufacturer's manual. RT-qPCR was conducted with the gene-specific primers and QuantiTect SYBR Green PCR Kit (QIAGEN) in Bio-Rad CFX96 Real-Time System (Bio-Rad). The primer pairs used for RT-qPCR are listed in the Table S1. Relative expression levels were calculated with three technique repeats by the $2^{-\Delta\Delta CT}$ method (Livak & Schmittgen, 2001). Statistical analysis was performed by one-way ANOVA or Student's *t*-test. Quantitative data ware processed by GraphPad Prism 7.0.

Methods S6 Pathogen inoculation and microscopy & analysis

Powdery mildew isolate *Golovinomyces cichoracearum* UCSC1 was maintained on the leaves of *pad4/sid2* double mutant plants. The method used for inoculation and quantification of disease susceptibility was conducted following a previous report (Zhao *et al.*, 2015). Briefly, 5-week-old plants were evenly inoculated with dislodged *G. cichoracearum* UCSC1 conidia that collected from the infected *pad4/sid2* mutant leaves using a brush. To analysis the number of the spores, representative inoculated leaves were collected at 10 days post inoculation (dpi) and washed in 3 ml sterile water containing 0.01% Tween 20 by shaking for 20 min at 200 rpm, the blood cell counting plate was used for counting the spores under microscope (Zeiss imager A2). Cell death and H_2O_2 accumulation in the leaves of 6-week-old plants were examined by trypan blue staining and 3,3'-diaminobenzidine (DAB) staining, respectively (Xiao *et al.*, 2003). Dying cells and the fungal structures in the inoculated leaves were analyzed by trypan blue staining at 10 dpi for powdery mildew according to the previously described methods (Xiao *et al.*, 2003). All the images were processed using Zeiss LSM Image Browser, GraphPad Prism and Adobe Photoshop or Image J.

Methods S7 Bacterial growth assays

For bacterial growth assay, 5-week-old *Arabidopsis* plants were used and infiltrated with the virulent strain *Pseudomonas syringae* pv. *tomato* DC3000 (*Pst* DC3000) at the concentration of

OD600 = 0.0005. Bacterial propagation was determined by colony counting as previously described (Li *et al.*, 2010) at 0 and 3 dpi, respectively.

Methods S8 Luciferase (LUC) reporter assays in *Nicotiana benthamiana*

For dual-luciferase reporter assay, the coding sequences of *ethylene response factor* (*ERF*) genes were amplified from the cDNA of Col-*gl* with the gene-specific primer pairs indicated in Table S1. The DNA fragments of each gene were cloned into the pCAMBIA1300 vector at *Kpn*I/*Spe*I sites, forming the effecter constructs. The full-length of *RPW8.1* promoter was amplified from the genomic DNA of R1Y4 with the primer pairs indicated in Table S1 and was cloned into pCAMBIA1300-*LUC* vector at the *Kpn*I site, forming reporter construct (named $Pro_{RPW8.1}$-*LUC*). *RLUC* (*Renilla luciferase*) driven by CaMV 35S was used as an internal control. *Agrobacteria* strains harboring recombinant plasmids were incubated in LB liquid medium containing kanamycin (50 μg mL^{-1}), rifampin (50 μg mL^{-1}) and gentamicin (50 μg mL^{-1}) at 28°C for overnight on a shaking platform (200 r min^{-1}). Subsequently, the bacteria were collected and adjusted to an OD600 value of 1 with MMA buffer (10 mM $MgCl_2$; 10 mM MES; 200 mM acetosyringone). Re-suspended *Agrobacteria* bacterial containing effecter and reporter were mixed in a 1:1 rate and co-infiltrated into *N. benthamiana* leaves. Total proteins were isolated using the Dual-Luciferase Reporter Assay System (Promega) following the manufacturer's manual two days after infiltration. LUC and RLUC activity were detected using the Dual-Luciferase Reporter Assay System (Promega) in GLOMAX96 Microplate Luminometer system (Promega) according to the manufacturer's manual. Six independent replicates were performed.

For LUC reporter assay, *Agrobacteria* strain harboring the individual effecter plasmid was co-infiltrated into *N. benthamiana* leaves with $Pro_{RPW8.1}$-*LUC*. All the images were taken using a low-light cooled CCD imaging apparatus two days after infiltration. The relative LUC fluorescence intensity were determined by Image J and normalized to eYFP control.

Methods S9 Yeast one-hybrid (Y1H) assays

Y1H was performed for DNA-protein binding assay. The coding sequences of *ERF*6, *ERF016* and *ORA59* were amplified from the cDNA of Col-*gl* with the gene-specific primer pairs listed in Table S1. The cDNA fragments of each gene were cloned into the pGADT7-Rec2 vector (Clontech) at *Ned*I/*Eco*RI sites, forming pGADT7-ERF6, pGADT7-ERF016 and pGADT7-ORA59,

respectively. The full-length and truncated fragment of the *RPW8.1* promoter were amplified from the genomic DNA of R1Y4 with the primer pairs indicated in Table S1 and were cloned into pHIS2 vector (Clontech) at the *Eco*RI/*Sac*I sites, forming the reporter vectors. Then, each pGADT7-ERF fusion construct was co-transformed with the reporter vector ($Pro_{RPW8.1}$-pHIS2, $Pro_{RPW8.1}$-pHIS2-I, $Pro_{RPW8.1}$-pHIS2-II, $Pro_{RPW8.1}$-pHIS2-III, $Pro_{RPW8.1}$-pHIS2-IV and $Pro_{RPW8.1}$-pHIS2-V) into the Y187 yeast cells according to the manufacturer's manual (Clontech), respectively. The pairs of empty vector pGADT7-Rec2 and pHIS2 expressing various lengths of RPW8.1 promoter were employed as negative controls. Yeast cells grown on the selective medium SD/-Trp/-Leu/-His supplemented with 90 mM 3-amino-1,2,4-triazole (3-AT) indicated the DNA-protein interactions.

Methods S10 Protein expression and purification

The coding sequences of *ORA59* were amplified from the cDNA of R1Y4 with the primer pairs indicated in Table S1 and were cloned into the pGEX-6p-1 vector at the *Bam*HI/*Eco*RI sites to generate *Glutathione-S-transferase* (*GST*): *ORA59*. *Escherichia coli* BL21 (DE3) harboring the fusion construct was grown in the LB medium containing 100 μg mL^{-1} ampicillin at 37 ℃ to OD600 = 0.5. The expression of the fusion protein GST-ORA59 was induced by 0.2 mM isopropyl β-D-1-thiogalactopyranoside (IPTG) and incubation at 16 ℃ for overnight. Glutathione-agarose beads (BD biosciences) was used for purifying GST-ORA59 protein according to the manufacturer's manual.

Methods S11 CRISPR/Cas9 plasmids construction and mutant screening

To generate *ERF6*, *ERF016* and *ORA59* knockout mutants, CRISPR (clustered regularly interspaced short palindromic repeats)/Cas9 plasmids of each gene were constructed as described previously (Wang *et al.*, 2015). Briefly, the guide RNA sequences in *ERF6*, *ERF016* and *ORA59* were screened and designed by *CRISPR-PLANT* system (https://www.genome.arizona.edu/crispr/CRISPRsearch.html), the special primers containing gene-specific spacer sequences were used and cloned into the binary vector pHEE401E at *Bsa*I site, resulting in knockout constructs. All the constructs were verified by sequencing and transformed into R1Y4 and Col-*gl* via *Agrobacterium* strain GV3101-mediated transformation. In order to confirm the genotype of knockout lines. Hygromycin was used for screening positive T1 plants. Then genomic DNA was extracted from hygromycin-positive lines using CTAB method,

PCR amplification was carried out using gene-special primers pairs. PCR products were sequenced to detect mutations. All the primers used in this study were listed in the Table S1.

References

Huang YY, Zhang LL, Ma XF, Zhao ZX, Zhao JH, Zhao JQ, Fan J, Li Y, He P, Xiao S, et al. 2019. Multiple intramolecular trafficking signals in RESISTANCE TO POWDERY MILDEW 8.2 are engaged in activation of cell death and defense. *THE PLANT JOURNAL* **98**(1): 55-70.

Li Y, Zhang Q, Zhang J, Wu L, Qi Y, Zhou JM. 2010. Identification of microRNAs involved in pathogen-associated molecular pattern-triggered plant innate immunity. *Plant Physiology* **152**(4): 2222-2231.

Li Z, Peng J, Wen X, Guo H. 2013. Ethylene-insensitive3 is a senescence-associated gene that accelerates age-dependent leaf senescence by directly repressing miR164 transcription in *Arabidopsis*. *The Plant cell* **25**(9): 3311-3328.

Livak KJ, Schmittgen TD. 2001. Analysis of relative gene expression data using real-time quantitative PCR and the 2− ΔΔCT method. *Methods (San Diego, Calif.)* **25**(4): 402-408.

Wang ZP, Xing HL, Dong L, Zhang HY, Han CY, Wang XC, Chen QJ. 2015. Egg cell-specific promoter-controlled CRISPR/Cas9 efficiently generates homozygous mutants for multiple target genes in *Arabidopsis* in a single generation. *Genome biology* **16**(1): 144.

Xiao S, Brown S, Patrick E, Brearley C, Turner JG. 2003. Enhanced transcription of the *Arabidopsis* disease resistance genes *RPW8.1* and *RPW8.2* via a salicylic acid–dependent amplification circuit is required for hypersensitive cell death. *The Plant cell* **15**(1): 33-45.

Yang C, Li W, Cao J, Meng F, Yu Y, Huang J, Jiang L, Liu M, Zhang Z, Chen X, et al. 2017. Activation of ethylene signaling pathways enhances disease resistance by regulating ROS and phytoalexin production in rice. *THE PLANT JOURNAL* **89**(2): 338-353.

Zhang B, Liu H, Ding X, Qiu J, Zhang M, Chu Z. 2017. AtACS8 plays a critical role in the early biosynthesis of ethylene elicited by copper ions in *Arabidopsis*. *Journal of Cell Science* **131**(2): jcs. 202424.

Zhao Z-X, Xu Y-B, Wang T-T, Ma X-F, Zhao J-Q, Li Y, Fan J, Wang W-M. 2015. Proper expression of AS1 is required for RPW8. 1-mediated defense against powdery mildew in *Arabidopsis*. *Physiological and Molecular Plant Pathology* **92**: 101-111.

Zhao ZX, Feng Q, Cao XL, Zhu Y, Wang H, Chandran V, Fan J, Zhao JQ, Pu M, Li Y, et al. 2019. *Osa-miR167d* facilitates infection of *Magnaporthe oryzae* in rice. *Journal of Integrative Plant Biology* **62**: 702-715.
